# Impacting the balance between CO_2_ and proton reduction by control over aggregation in a model π-conjugated N-heterocycle – proflavine†

**DOI:** 10.1039/d5sc06873h

**Published:** 2026-05-15

**Authors:** Yana Reva, Jonas Färber, Yifan Bo, Maximilian A. Thiele, Christian Hanke, Ayşe Günay-Gürer, Maximillian Herm, Johannes A. C. Barth, Dirk M. Guldi

**Affiliations:** a Department of Chemistry and Pharmacy, Profile Center FAU Solar, Interdisciplinary Center for Molecular Materials (ICMM), Friedrich-Alexander-Universität Erlangen-Nürnberg 91058 Erlangen Germany dirk.guldi@fau.de; b Department Geographie und Geowissenschaften, Geozentrum Nordbayern, Friedrich-Alexander-Universität Erlangen-Nürnberg (FAU) Schlossgarten 5 91054 Erlangen Germany

## Abstract

In this study, we investigated the mechanistic factors that govern the selective photocatalytic reduction of CO_2_ over protons within a simple π-conjugated N-heterocycle, proflavine. Diluted conditions, where aggregates of 65 nm are formed, favored the selective CO_2_ photo-reduction, while the gradual transition to concentrated conditions enabled photo-reductive H_2_ generation. Proton reduction is coupled to larger aggregates, in which an alternative photo-relaxation pathway is active. We used transient absorption spectroscopy to corroborate that at low proflavine concentrations the presence of an electron donor triggers the one-electron reduced proflavine to perform the direct CO_2_ reduction. At high proflavine concentrations, protonation of the one-electron reduced proflavine was favored due to a positive shift in basicity in larger aggregates with sizes over 1 µm. In turn, H_2_ abstraction began with a pair of one-electron reduced, protonated, intermediates. Our study demonstrates an effective approach to limiting water reduction, a key challenge in advanced metal-free organic photocatalysis.

## Abstract 2

Photoreduced proflavine acts as a photocatalyst, vastly yielding CO_2_ reduction products. Exceeding a critical concentration leads to increased clustering and H_2_ formation supersedes, due to increased basicity of the reduced, aggregated species.

## Graphical abstract

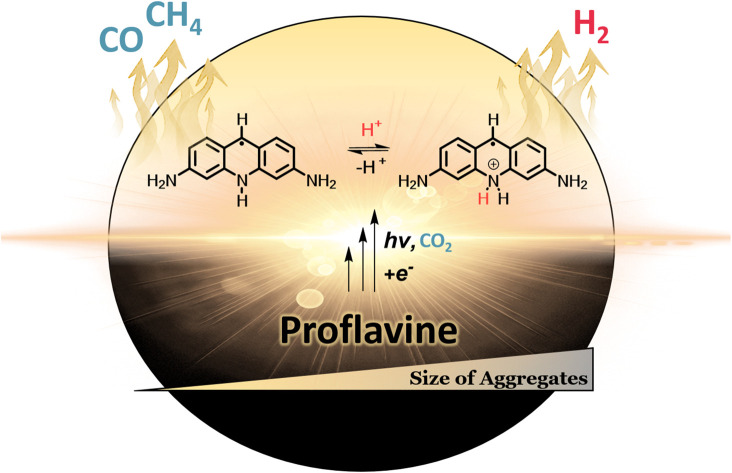

## Introduction

Solar light-driven photocatalytic reduction of carbon dioxide (CO_2_) in water has been demonstrated to be a promising pathway for sustainable energy conversion with the potential to reduce greenhouse gas emissions.^1^ Despite significant progress in the field over the years, groundbreaking research continues to face emerging challenges. Competing water reduction, high prices for gas separation of valuable carbon-material from dihydrogen, and CO_2_ recycling efficiency must be addressed.^2^ For instance, to circumvent post-catalytic gas separation, the development of highly selective photocatalysts for CO_2_ reduction is essential.

In photo-redox reactions, photocatalysts are excited, subsequently reduced by a suitable electron donor to finally produce a catalytically active species, that reduces the substrate by direct electron transfer.^3,4^ The primary activation is crucial and involves bending of CO_2_ and overcoming the negative reduction potential of −1.9 V *vs.* NHE.^5^ Chemisorptive activation appears essential to lower the activation barrier and facilitate CO_2_ reduction.^6^ By tuning the electron-donating strength, light-activated reduction of the photocatalyst governs both its interactions with CO_2_ and the subsequent substrate reduction. In contrast, protonation of the reduced photocatalyst deactivates the CO_2_ reduction pathway, lowers its electronegativity, and creates favorable conditions for proton reduction and, therefore, H_2_ generation.^7^

With a focus on the competing CO_2_ and H^+^ reduction, any change in the photocatalyst concentration commonly affects both pathways in the same way.^8–10^ The scenario changes, however, as soon as aggregation comes into play. In metal-based photocatalysts, aggregation enhances the catalytic CO_2_ reduction efficiency, while minimizing the competing water reduction.^11,12^ A similar balance in organic, metal-free catalysts has not yet been extensively investigated, highlighting a significant knowledge gap. The intrinsic proton affinity of organic materials and p*K*_a_ shifts in aggregated systems^13–15^ collectively suggest that aggregation is crucial for determining the photocatalytic selectivity. One prominent example of an efficient metal-free CO_2_ reducing photocatalyst is covalent organic frameworks (COFs). COF's selective photo-reduction of CO_2_ in aqueous solutions using visible light is remarkable.^16–18^ COFs, which are constructed from π-conjugated N-heterocycles, are of particular interest here. For instance, Fu *et al.* found, that metal-free triazine-based COFs drive a clear-cut photocatalytic conversion of CO_2_ to methanol, producing only trace amounts of H_2_.^19^ Hereby, π-conjugated N-atoms, which serve as photocatalytic centers in the triazine-building blocks,^20^ play a crucial role.

In this work, we address the nuanced interplay between aggregation and selectivity in the reduction of CO_2_ over protons. The model system proflavine – a π-conjugated N-heterocycle was utilized. Considering both, the ability to form aggregates^21^ and a suitable pH window to tune the de-/protonation of the active photoreduced species,^22,23^ proflavine represents an excellent model.

**Fig. 1:**
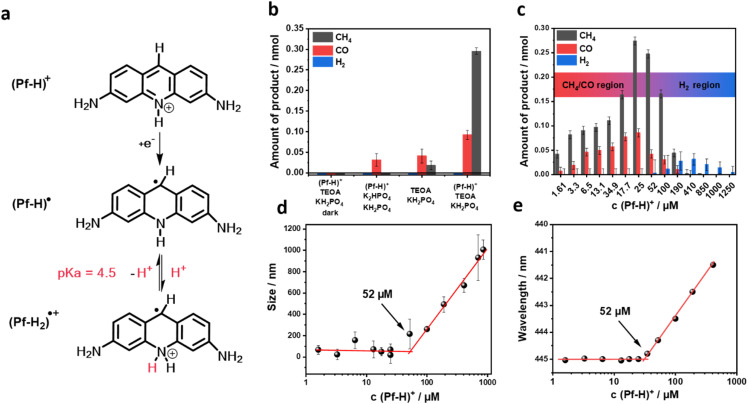
(a) Relevant structures. (b) Rate of CH_4_, CO, and H_2_ evolution for references at pH 7 after 1 h upon 1 sun irradiation with a Xe-lamp equipped with 1.5 AM filter. (c) Rate of CH_4_, CO, and H_2_ evolution measured in aqueous solution of 4.28 vol% TEOA, 0.55 M KH_2_PO_4_, at pH 7, and with different concentrations of (Pf-H)^+^ over 1 hour of irradiation. (d) DLS analysis in aqueous solution of 4.28 vol% TEOA, 0.55 M KH_2_PO_4_, at pH 7, and with different concentrations of (Pf-H)^+^. (e) Concentration-dependent shift in absorbance of aqueous solutions with 4.28 vol% TEOA, 0.55 M KH_2_PO_4_, and at pH 7.

In the ground state at pH 7, proflavine exists primarily in its single protonated form, as (Pf-H)^+^ (Fig. 1a).^24–28^ Following photoexcitation at 443 nm and intersystem crossing (ISC) under deoxygenated conditions, the long-lived triplet excited state (T_1_)(Pf-H)^+^ emerges.^27,29^ In the presence of an electron donor, such as TEOA, (T_1_)(Pf-H)^+^ with an energy of 2.17 eV accepts an electron and is transformed into the reduced (Pf-H)˙ (Fig. 1a). Because of its reduction potential of −1.57 V *vs.* SHE (Pf-H)˙ is active in numerous photo-redox reactions.^22,24,30–33^ The reduced species exhibits a p*K*_a_ value of 4.5 and, therefore, forms (Pf-H_2_)˙^+^ under acidic conditions (Fig. 1a).^23^ Noticeably, electron-rich and conjugated nitrogen atoms are present. Thus, we hypothesise that (Pf-H)˙ chemisorbs CO_2_ at the electron-deficient carbon site and then facilitates its reduction. Additionally, aggregate formation is thought to be beneficial for altering its proton affinity, a key step for protonation and thus for H_2_ generation.

## Results and discussion

### Photocatalysis

N-conjugated organic metal-free photocatalysts commonly generate single-carbon compounds like CO, HCOOH, HCHO, CH_3_OH, and CH_4_, with a maximum of 8 transferred electrons.^16–20^ The photocatalytic CO_2_ reduction was performed with 25 µM (Pf-H)^+^ in aqueous solutions containing 4.28 vol% TEOA and 0.55 M KH_2_PO_4_ at a pH of 7. The reaction mixture was first saturated with high purity CO_2_, minimizing O_2_ contamination, and then irradiated with a Xe-lamp using a 1.5 AM filter (1 sun). Under these conditions, gas-phase analysis after 1 h of irradiation afforded 0.1 nmol CO, 0.3 nmol CH_4_, and no H_2_ (Fig. 1b).§§The corresponding values were further used as references and subtracted to reveal the amount of photocatalytically generated products. When omitting either the electron donating TEOA or the buffering KH_2_PO_4_ or keeping non-irradiated samples, only traces of CH_4_ and CO were discernible. These results demonstrate the successful photocatalytic reduction of CO_2_ mediated by proflavine.¶¶The reported product yields are not normalized by turnover number (TON) due to the observed degradation of the photocatalyst over time. This instability implies that the catalyst's activity diminishes throughout the reaction period, making traditional TON calculations unrepresentative of the system's true catalytic efficiency. To optimize the catalytic conditions, the pH was varied between 6 and 8.5 by altering the KH_2_PO_4_ concentration. Hereby, a pH of 7 was found to deliver the maximum amount of CH_4_ and CO (Fig. S1). Next, we changed the (Pf-H)^+^ concentrations and examined the photocatalysis during an illumination period of one hour (Fig. 1c). The selected concentration range was given by the absence of discernible products. At a (Pf-H)^+^ concentration of 25 µM, the highest amounts of CH_4_ and CO were monitored. Notably, no other products such as HCOOH, HCHO, or CH_3_OH were detected under such optimized conditions (Fig. S2). Of great interest is the fact that at (Pf-H)^+^ concentrations larger than 200 µM, the generation of H_2_ dominated that of CH_4_ and CO. For example, at 410 µM, only traces of CH_4_ are detectable, while H_2_ levels reached a maximum of 3.25 nmol h^−1^. At 1000 µM, photocatalysis only yields H_2_ without any significant amount of CH_4_ or CO. The lack of any CO_2_ formation at high (Pf-H)^+^ concentrations proves that none of the photo-products, that is, CH_4_ or CO, stem from the photo-decomposition of the catalyst. Additionally, we compared two identical photocatalytic systems, both containing 25 µM (Pf-H)^+^, at the optimized photocatalytic conditions. One system was purged with CO_2_ and the other with O_2_ (supporting superoxide-mediated decomposition) prior to irradiation. The O_2_-purged sample failed to generate any detectable CH_4_ after one hour of irradiation. Conclusively, the observed CH_4_ is produced exclusively in the photocatalytic reduction of CO_2_ by proflavine (Fig. S3). The competition between CO_2_ and H^+^ reduction must be influenced by the intrinsic properties of the catalyst. This conclusion is further supported by the selective CO_2_ reduction occurring even in solutions at high proton concentrations.

Considering the aforementioned results, we not only infer two different photo-relaxation pathways, but also their dependence on the photocatalyst concentration. To elaborate on the nature of the concentration-dependent CO_2_/H^+^ reduction selectivity, dynamic light scattering (DLS) was measured under optimized photocatalytic conditions, with a (Pf-H)^+^ concentration ranging from 1.6 to 850 µM (Fig. 1d). At concentrations below 52 µM, (Pf-H)^+^ forms aggregates with an average size of 65 nm in the presence of 4.28 vol% TEOA and 0.55 M KH_2_PO_4_ (pH 7). At 52 µM dihydrogen is generated and the aggregate size notably increases. At 1000 µM, where photocatalysis yields solely H_2_, the average size of the aggregates reached 1 µm. The absorption spectra of (Pf-H)^+^ across different concentrations were examined (Fig. S4). Starting at around 52 µM, a stepwise blue-shift in the absorption maximum from 445 to 441 nm along with the emergence of a shoulder at 482 nm were observed. This supports the notion that aggregates are growing when a critical concentration of (Pf-H)^+^ is passed (Fig. 1e). To this end, photocatalytic tests using 1000 µM (Pf-H)^+^ in aqueous solutions containing 4.28 vol% TEOA were conducted and the pH was changed from 6.5 to 10 by varying KH_2_PO_4_ (Fig. S5). Importantly, below a pH of 7 all photo-products relate to the H^+^ reduction. Going beyond a pH of 7, CO_2_ reduction products were found next to those stemming from H^+^ reduction.||||The concentration of H_2_ has remained constant. Aggregation has caused a shift in the p*K*_a_ of the active species, (Pf-H)˙. A stronger basicity facilitates the protonation, on one hand, and enhances H_2_ generation, on the other hand.

### CO_2_ reduction mechanism

The reduced form of proflavine, (Pf-H)˙, was prepared by chemically reducing (Pf-H)^+^ with LiAlH_4_. The product solution was then filtered and the pH adjusted to 7 by adding hydrochloric acid. An important fingerprint of (Pf-H)˙ is its 394 nm absorption (Fig. S6).

Further, mass spectrometry with the chemically generated (Pf-H)˙ was conducted and a mass of 209.01 *m*/*z* was found in the negative ion mode. This is in good agreement with the corresponding isotope pattern (Fig. S7).**Storing a (Pf-H)˙ solution for 24 h confirmed its inertness in water (Fig. S8). Additionally, a titration of (Pf-H)˙ under inert atmosphere corroborates the 446 nm fingerprint of protonation below a pH of 4.5. Corresponding titration curves and spectra are all gathered in Fig. S9. In another experiment, we saturated a (Pf-H)˙ solution with CO_2_ and noted a red-shift of the absorption to 443 nm. Implicit is the re-oxidation of (Pf-H)˙ and regeneration of (Pf-H)^+^ (Fig. S10). Moreover, the gaseous products generated upon CO_2_ saturation of (Pf-H)˙, namely CO and CH_4_, were analysed. This data was compared with that recorded for a CO_2_-saturated solution of (Pf-H)^+^ at 25 µM, which lacked any CO_2_ reduction (Fig. S11). (Pf-H)˙ showed a striking kinetic selectivity: while it exhibited high stability against re-oxidation by O_2_ (only 2% re-oxidized after 24 hours in 99.9% O_2_), it underwent full and instant re-oxidation when exposed to CO_2_, confirming the CO_2_ pathway's kinetic preference (Fig. S12). Given the absence of an electron donor, buffer or photoirradiation, CO and CH_4_ unambiguously stem from the direct CO_2_ reduction. Hence, we postulate that (Pf-H)˙ is a key species in the photocatalytic CO_2_ reduction.

To provide unambiguous evidence for the carbon conversion pathway, we monitored the ^13^C/^12^C ratios in both CO_2_ and CH_4_ using wavelength-scanned cavity ring-down spectroscopy. The ^13^C/^12^C ratios are expressed in a delta notation as a permille deviation from Vienna Pee Dee Belemnite (*i.e. δ*^13^C in ‰ VPDB) as standard. They were determined for CO_2_ and CH_4_ in the complete photocatalytic system and solutions in the absence of an electron donor, buffer or photoirradiation (Table S1).††††The increased concentration of CH_4_ in the isotopic measurements are explained, as here a different setup was used, which demanded a decreased gas to liquid phase ratio in the containers paired with an increased irradiation surface of the vessels. The originally added CO_2_ had a *δ*^13^C value of −41.6‰. It was only upon CO_2_ addition together with all other additives that we found significant CH_4_ yields. Under these conditions, *δ*^13^C_CO_2__ decreased by a maximum of 7.0‰. At the same time, any detectable CH_4_ was around an average of −37.3‰. From these findings we conclude that CH_4_ stems from CO_2_ reduction with a preferential uptake of ^13^C. Additionally, the ^13^C/^12^C ratios for any dissolved organic carbon (doc) were measured by isotope ratio mass spectrometry with and without irradiation (Table S2). Our *δ*^13^C_doc_ measurements lacked any deviations, but differed noticeably from the *δ*^13^C of generated CH_4_. Negligible differences in *δ*^13^C_doc_ – with and without irradiation – coupled with their significant deviations from *δ*^13^C_CH_4__ confirms that CH_4_ is not a by-product of any organic material decomposition.

To gather additional information on the photoreduction mechanism proflavine was deposited onto a platinum surface and electrochemical *operando* Raman spectroscopy was conducted. The aqueous phase was purged with CO_2_ and KH_2_PO_4_/K_2_HPO_4_ were added to adjust the pH to 7. Broad fingerprints between 750 and 1750 cm^−1^ were taken as evidence for the proflavine stability at 0 V *vs.* Ag/AgCl. Under reductive conditions, that is, −1.5 V *vs.* Ag/AgCl, new features emerged as evident from the corresponding differential spectrum. Compared to reference measurements at 0 V *vs.* Ag/AgCl, the spectral changes at −1.5 V *vs.* Ag/AgCl revealed broad signals with distinguishable peaks around 1307 and 2208 cm^−1^, corresponding to symmetric –C–O stretching vibration of a carboxylate-like species (CO_2_ adsorption) and –C≡O stretching vibration (CO adsorption), respectively (Fig. S13). These features are the crucial intermediates in the formation of CO and CH_4_. Their presence confirms the successful adsorption and subsequent reduction of CO_2_ by proflavine.

### Photodegradation

Proflavine is known to undergo side reactions during photocatalysis.^27^ To demonstrate, that such side reactions have minimal impact on the product selectivity, 25 µM (Pf-H)^+^ were photoirradiated in the presence of 4.28 vol% TEOA and 0.55 M KH_2_PO_4_ for four hours rather than one (Fig. S14).‡‡‡‡The gaseous phase was analyzed and the sample was re-purged with CO_2_ every 30 min. In parallel, the absorption spectrum of the liquid phase was monitored (Fig. S15). CO and CH_4_ increase during the photoirradiation. Importantly, no appreciable H_2_ was detected. It is, however, noted that after three hours, the generation rates of the products plateaued and decayed, likely due to the disproportionation of (Pf-H)^+^. A continuous decrease of the 445 nm absorption and the concurrent formation of dihydroproflavine and leuco-proflavine with their characteristic absorptions at 295 and 340 nm, respectively, validates the disproportionation.^27^ Based on these findings, we demonstrated that the product selectivity is not impacted by the formation of any side products.

### Excited state spectroscopy at low concentrations – 65 nm aggregates

First, fs-TA for an aqueous buffered solution (with 0.5 M K_2_HPO_4_/KH_2_PO_4_) of 25 µM (Pf-H)^+^ without any electron donor was considered. fs-TA spectra were deconvoluted with a kinetic model based on two species and two evolution-associated spectra (EAS), that is, EAS-1 and EAS-2 (Fig. S16), were obtained.

EAS-1, for which a lifetime of 3.8 ps was determined, showed excited state absorptions (ESAs) at 400 nm, ground state bleaching (GSB) at 445 nm, and stimulated emission (SE) at 515 nm. All of them are in excellent agreement with the steady state absorption and fluorescence spectra of (S_0_)(Pf-H)^+^ (Fig. S17). As the decay of EAS-2 is outside the fs-TA timescale, we conducted additional ns-TA experiments. ns-TA spectra were also fitted obtaining two EASs, that is, EAS-2 and EAS-3 (Fig. S18). The characteristics of EAS-2, which include ESA at 400 nm, GSB at 445 nm, and SE at 515 nm, are like those of EAS-1. From the similarity between EAS-1 and EAS-2 it was concluded, that the two are a vibrationally hot singlet excited state (S^hot^_1_)(Pf-H)^+^ and a vibrationally relaxed singlet excited state (S^rel^_1_)(Pf-H)^+^, respectively. After 4.7 ns, SE is replaced by ESAs < 390 nm and between 500 and 750 nm. These comprise EAS-3 and feature a lifetime of 29.2 µs. In line with sensitization experiments using (Ir[dF(CF_3_)ppy]_2_(dtbpy))PF_6_ as a triplet sensitizer,^34^ EAS-3 is attributed to the triplet excited state – (T_1_)(Pf-H)^+^.§§§§ns-TA in oxygen-free methanol solutions of (Ir[dF(CF_3_)ppy]_2_(dtbpy))PF_6_ and (Pf-H)^+^ resulted in a 1.38 µs lasting formation of ESAs < 390 and between 500 and 750 nm that decayed with 28.2 µs. All reference experiments are summarized in Fig. S19–S21.

**Fig. 2:**
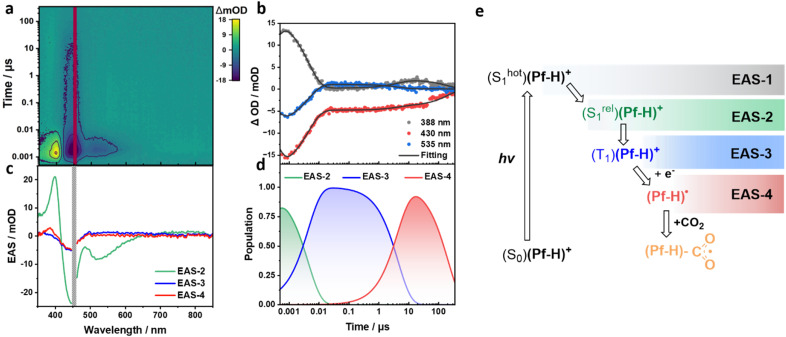
(a) ns-TA heat map of 25 µM (Pf-H)^+^ in aqueous solutions with 4.28 vol% TEOA and 0.55 M KH_2_PO_4_. The photoexcitation wavelength was set to 450 nm. (b) Representative time traces at 388, 430, and 535 nm depicting the recorded evolution of the transients. (c) Evolution associated spectra (EAS) from the sequential three-exponential global analyses of the ns-TA spectra. EAS-2, EAS-3, and EAS-4 correspond to (S^rel^_1_)(Pf-H)^+^ with a lifetime of 4.5 ns, (T_1_)(Pf-H)^+^ with a lifetime of 4.5 µs, and (Pf-H)˙, respectively. (d) Time profiles depicting the time-resolved population of the corresponding EASs from global analyses of the ns-TA spectra. (e) Corresponding mechanistic cascade of transient states.

In the next set of our experiments TEOA was added: 25 µM (Pf-H)^+^, 0.55 M KH_2_PO_4_, and 4.28 vol% TEOA. On the fs-TA timescale, the only fully resolvable EAS was that of EAS-1 with 450 nm GSB and 515 nm SE. It is the (S^hot^_1_)(Pf-H)^+^ (Fig. S22). Its lifetime is with 7.5 ps slightly longer than the reference experiments. On the ns-TA timescale, the data was taken and best deconvoluted when using a three species kinetic model (Fig. 2). EAS-2 is also like in the experiments without TEOA – albeit being lower in intensity – identified as (S^rel^_1_)(Pf-H)^+^. It takes 4.5 ns, by which EAS-2 gives place to EAS-3, namely a localized (T_1_)(Pf-H)^+^. Notably, (T_1_)(Pf-H)^+^ is in the presence of TEOA shorter-lived than in the absence of TEOA; 4.5 *versus* 29.2 µs.

To gather insights into the TEOA-induced quenching of (Pf-H)^+^, we conducted ns-TA at different TEOA concentrations. The pH was kept constant at 7 by means of adjusting the KH_2_PO_4_ concentrations. Results were quantitatively treated with the Stern–Volmer relationship, *τ*_0_/*τ* = 1 + *K*_SV_[TEOA], in which *K*_SV_ is the Stern–Volmer constant, [TEOA] is the concentration of TEOA, and *τ*_0_ and *τ* are the (T_1_)(Pf-H)^+^ lifetimes in the absence and presence of TEOA, respectively. The linear relationship was ascribed to the dynamic electron-transfer quenching of (T_1_)(Pf-H)^+^ by TEOA.¶¶¶¶In order to reach pH 7, the TEOA-free sample was buffered with KH_2_PO_4_ and K_2_HPO_4_. Different electrolyte-composition causes an offset of the *τ*_0_ value, deviating the linear behavior and, therefore was excluded from fitting. With the *K*_SV_ of 16.4 M^−1^ the bimolecular quenching rate constant (*K*_q_) was calculated to be 5.6 × 10^5^ M^−1^ s^−1^ (Fig. S23). A low *K*_q_ is attributed to the partial protonation of TEOA at pH 7 due to a p*K*_a_ value of 7.76, lowering the concentration of its unprotonated, electron-donating form. Therefore, the presence of protonated TEOA limits the rate constant well below the diffusion-controlled limit.

The quenching of (T_1_)(Pf-H)^+^ by the electron donating TEOA resulted in EAS-4, for which a distinct 388 nm ESA and a lifetime outside the time-range of our ns-TA set-up were derived. Considering a significant spectral overlap of the differential (Pf-H)˙ − (Pf-H)^+^ and EAS-4 as well as the presence of an electron-donating TEOA, EAS-4 is assigned to (Pf-H)˙ (Fig. S24). Fig. 2e summarizes the proposed mechanism for the low concentration experiments – aggregate size 65 nm.

To exclude any impact of TEOA degradation products onto the proposed mechanism, ns-TA was conducted with 25 µM (Pf-H)^+^ in water, using 4.28 vol% methanol rather than TEOA as electron donor. The raw data was deconvoluted with a kinetic model based on three species (Fig. S25). All three EASs match those discussed when employing electron donating TEOA and, in turn, prove its electron donation.

### Excited state spectroscopy at intermediate concentrations – 700 nm aggregates

A systematic increase in (Pf-H)^+^ concentration alters the product distribution from an exclusive CO/CH_4_ mixture to one also featuring H_2_ (Fig. 1c). Mechanistic aspects were investigated by using aqueous solutions of pH 7 with 410 µM (Pf-H)^+^, 0.55 M KH_2_PO_4_, and K_2_HPO_4_.

Global analyses of fs-TA reveal two consecutively emerging EAS, that is, EAS-1 and EAS-2. They correspond to (S^hot^_1_)(Pf-H)^+^ and (S^rel^_1_)(Pf-H)^+^ and match the spectral characteristics seen in the low concentration regime (Fig. S26). It takes 3.2 ps for (S^hot^_1_)(Pf-H)^+^ to interconvert into (S^rel^_1_)(Pf-H)^+^. To determine the lifetime of EAS-2 we conducted ns-TA. Interpretation of the 410 µM (Pf-H)^+^ ns-TA experiments required a kinetic model based on three species, that is, EAS-2, EAS-3, and EAS-4 (Fig. S27). EAS-2 are similar on the fs- and ns-time scales. The lifetime of (S^rel^_1_)(Pf-H)^+^) is 5 ns. EAS-3 and EAS-4 exhibit similar spectral properties: ESAs < 390 nm and between 500 and 750 nm. To this end, EAS-3 and EAS-4 both are attributed to (T_1_)(Pf-H)^+^s; one short-lived with a lifetime of 1 µs, and one long-lived with a lifetime of 33 µs. We hypothesize that the 1 µs component of (T_1_)(Pf-H)^+^ is due to triplet–triplet annihilation and that it is activated by the high (Pf-H)^+^ concentration.

fs-TA spectroscopy of the TEOA-containing aqueous system with 410 µM (Pf-H)^+^, 4.28 vol% TEOA, and 0.55 M KH_2_PO_4_ gives rise to two EASs. For EAS-1, 400 nm ESA and 440 nm GSB are complemented by 515 nm SE (Fig. S28). This (S^hot^_1_)(Pf-H)^+^ lives for 33.3 ps and undergoes relaxation to afford (S^rel^_1_)(Pf-H)^+^. Insights into the decay of (S^rel^_1_)(Pf-H)^+^ came from ns-TA measurements. Fitting the ns-TA spectrum requires the use of four species rather than three like in the absence of TEOA.||||||||The corresponding time-trace fits and singular value decompositions of the residual matrices corroborate the choice of four (Fig. S29).

**Fig. 3:**
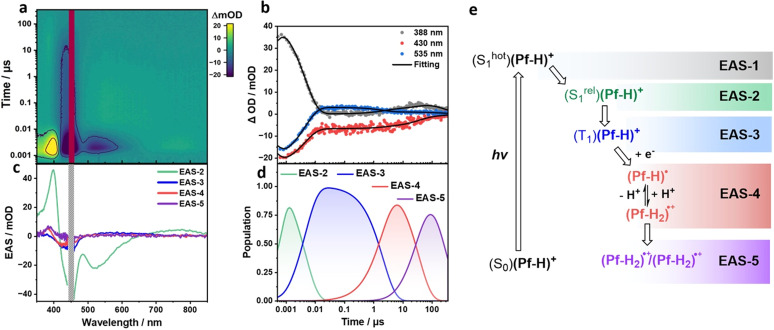
(a) ns-TA heat map of 410 µM (Pf-H)^+^ in aqueous solutions with 4.28 vol% TEOA and 0.55 M KH_2_PO_4_. The photoexcitation wavelength was set to 450 nm. (b) Representative time traces at 388, 430, and 535 nm depicting the recorded evolution of the transients. (c) Evolution associated spectra (EAS) from the sequential four-exponential global analyses of the ns-TA spectra. EAS-2, EAS-3, EAS-4, and EAS-5 correspond to (S^rel^_1_)(Pf-H)^+^ with a lifetime of 4.5 ns, (T_1_)(Pf-H)^+^ with a lifetime of 2.1 µs, [(Pf-H)˙ ↔ (Pf-H_2_)˙^+^] with a lifetime of 35.5 µs, and (Pf-H_2_)˙^+^/(Pf-H_2_)˙^+^, respectively. (d) Time profiles depicting the time-resolved population of the corresponding EASs from global analyses of the ns-TA spectra. (e) Corresponding mechanistic cascade of transient states.

For EAS-2, once again, 400 nm ESA, 440 nm GSB, and 515 nm SE due to (S^rel^_1_) are noted. (S^rel^_1_) undergoes intersystem crossing within 4.5 ns to afford (T_1_)(Pf-H)^+^ in the form of EAS-3 based on ESAs < 390 nm and between 500 and 750 nm (Fig. 3). Rather than undergoing ground state recovery as in the case of TEOA-free conditions, (T_1_)(Pf-H)^+^ decays quickly in the presence of 4.28 vol% TEOA within 2.1 µs. A 35.5 µs lived EAS-4 evolves from the interaction of (T_1_)(Pf-H)^+^ with the electron donating TEOA. The most prominent ESAs are discernible at 388 and 450 nm. As shown previously, (Pf-H)˙ protonation causes a rise of a 446 nm feature, which is assigned to (Pf-H_2_)˙^+^. To deconvolute the spectral signature of EAS-4, EAS-3 was subtracted from EAS-4. The resulting differential spectrum combines the spectral features of (Pf-H)˙ next to those of (Pf-H_2_)˙^+^ (Fig. S30). The final species, namely EAS-5, is an amplification of the 446 nm ESA and, therefore corresponds to a second (Pf-H_2_)˙^+^. Its lifetime is outside of the time-range covered by our ns-TA set-up. In stark contrast to the experiments at low (Pf-H)^+^ concentrations, protonation of (Pf-H)˙ and formation of (Pf-H_2_)˙^+^ are observable on the microsecond timescale, due to an increase in proton affinity of the reduced proflavine in the aggregates. Evidently, at the given concentrations a competing photocatalytic path evolves. A somewhat broader size distribution of aggregate sizes at the concentration of 410 µM renders the deconvolution of (Pf-H)˙ and (Pf-H_2_)˙^+^ and detection thereof impossible.****The model fitting with five species does not sufficiently resolve (Pf-H)˙ and (Pf-H_2_)˙^+^ separately (Fig. S31).Fig. 3e summarizes the proposed mechanism for intermediate concentrations of proflavine (700 nm-aggregate).

### Excited state spectroscopy at high concentrations – 1 µm-aggregates

**Fig. 4:**
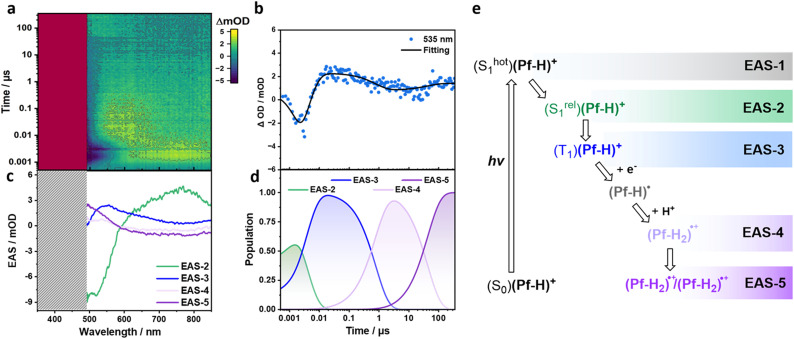
(a) ns-TA heat map of 1000 µM (Pf-H)^+^ in aqueous solutions with 4.28 vol% TEOA and 0.55 M KH_2_PO_4_. The photoexcitation wavelength was set to 450 nm. (b) Representative time trace at 535 nm depicting the recorded evolution of the transients. (c) Evolution associated spectra (EAS) from the sequential four-exponential global analyses of the ns-TA spectra. EAS-2, EAS-3, EAS-4, and EAS-5 correspond to (S^rel^_1_)(Pf-H)^+^ with a lifetime of 3.6 ns, (T_1_)(Pf-H)^+^ with a lifetime of 3 µs, (Pf-H_2_)˙^+^ with a lifetime of 43.6 µs, and (Pf-H_2_)˙^+^/(Pf-H_2_)˙^+^, respectively. (d) Time profiles depicting the time-resolved population of the corresponding EASs from global analyses of the ns-TA spectra. (e) Corresponding mechanistic cascade of transient states.

Given the overlapping absorbance of (Pf-H)^+^ and (Pf-H_2_)˙^+^, a clear differentiation between GSB and the evolution of (Pf-H_2_)˙^+^ mandates additional investigations. To this end, we considered a (Pf-H)^+^ concentration that would shift the photo-activity to the exclusive H_2_ formation. As such, fs-TA of aqueous solution with 1000 µM (Pf-H)^+^, 4.28 vol% TEOA, and 0.55 M KH_2_PO_4_ was recorded. Global fitting revealed two EASs upon sequential deconvolution. EAS-1 with a SE at 515 nm is assigned to (S^hot^_1_)(Pf-H)^+^ and lives for 63 ps (Fig. S32). EAS-2 outlives the ps-timescale. Therefore, we proceeded with ns-TA and used a global fit with a kinetic model based on four species (Fig. 4). After 3.6 ns, EAS-2 with the spectral fingerprints of (S^rel^_1_)(Pf-H)^+^ is replaced by EAS-3 with an intrinsic lifetime of 3 µs. EAS-3 is characterized by ESAs between 500 and 750 nm, which are the known (T_1_)(Pf-H)^+^ characteristics.

Following its 3 µs lasting decay, ESAs > 500 nm of EAS-4 are formed as (Pf-H_2_)˙^+^ grows. For the latter, a lifetime of 43.6 µs was derived. Notably, it was impossible to deconvolute (Pf-H)˙ found at the lower concentrations. We rationalize this fact by a p*K*_a_-driven shift towards (Pf-H_2_)˙^+^ rather than (Pf-H)˙.

Subsequently, EAS-5 evolves and is linked to the intensification of the ESA > 500 nm. EAS-5 lifetime extends beyond the temporal window of our ns-TA set-up. While the high (Pf-H)^+^ concentration of 1000 µM leads to strong ground-state absorbance that obscures the near-UV region, the emergence of the (Pf-H_2_)˙^+^ ESA is still evident, and its gradual intensification is clearly supported. Consequently, after the initial (Pf-H_2_)˙^+^ is produced, a second (Pf-H_2_)˙^+^ forms.

Our previous investigations with N-heterocyclic phenazine aggregates demonstrated that TEOA facilitated the formation of the second reduced and protonated phenazine *via* a chemical reduction that is linked to TEOA's self-oxidation reaction.^7^ In ns-TA, an intensification of the characteristic ESAs reflects the second reduction and protonation. Seeing a similar behavior in proflavine-aggregates, we conclude that the reduction of (T_1_)(Pf-H)^+^ and the formation of (Pf-H)˙ is linked to a positive p*K*_a_ shift in the 1 µm-aggregates. This facilitates protonation to afford (Pf-H_2_)˙^+^. (Pf-H_2_)˙^+^ subsequently reacts with a second (Pf-H_2_)˙^+^ before H_2_ is released. Fig. 4e summarizes the proposed mechanism for high concentrations of proflavine – aggregate size of 1 µm.

## Conclusions

We demonstrated that the degree of clustering functions as a molecular ruler that regulates the balance between photocatalytic CO_2_ and proton reduction in organic π-conjugated N-heterocyclic compounds, a consideration not previously reported in the literature. In this context, under dilute conditions, that is, small aggregate clusters, (Pf-H)^+^ photocatalysis predominantly yields CO_2_ reduction products. Proven by means of fs-TA and ns-TA spectroscopy, the addition of an electron donor enables the photo-induced formation of (Pf-H)˙, that is, the reduced form of (Pf-H)^+^. (Pf-H)˙ is, on one hand, stable in the absence of CO_2_ and, on the other hand, the catalytically active species that performs CO_2_ reduction. For example, exposing a solution of chemically reduced (Pf-H)˙ to CO_2_ resulted in its full re-oxidation together with CO and CH_4_ generation.

**Fig. 5:**
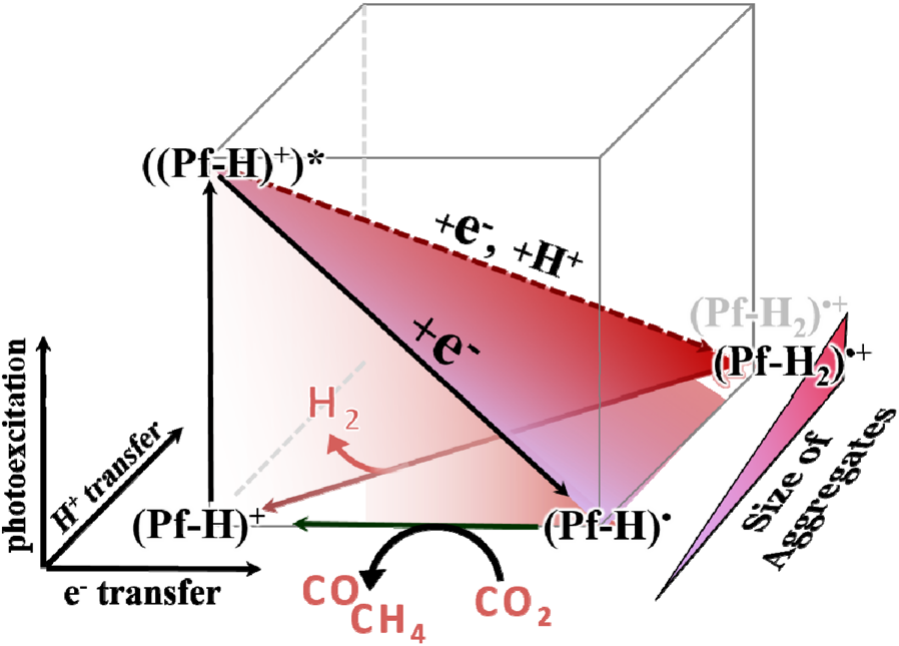
Electronic ground- and excited state square scheme in proflavine at pH 7 in the presence of electron donating TEOA.

Once the critical concentration is passed, the formation of H_2_ starts to compete with the CO_2_ reduction. This concentration represents a threshold beyond which the degree of clustering gradually increases. Based on fs-TA and ns-TA spectroscopy larger aggregates enforce the protonation of (Pf-H)˙, yield (Pf-H_2_)˙^+^, and allow for H_2_ release upon reaction with a second (Pf-H_2_)˙^+^. This reactivity is attributed to a positive shift in the p*K*_a_ of reduced proflavine at higher degrees of clustering. The intricate nature of this aggregation process provides a fertile ground for further investigation, opening up new avenues for understanding and controlling the mechanism of organic photocatalysts. Fig. 5 summarizes the complete mechanism.

## Author contributions

Y. R.: conceptualization of the involved mechanism, writing – original draft. Y. R. and J. F.: formal analysis, investigation of the photocatalytic activity and the involved photorelaxation cascade, data validation and visualization. M. A. T. and A. G.-G. confirmed absence of liquid-phase CO_2_ reduction products. J. F. Y. B. and M. H.: mechanism validation. M. A. T.: formal analysis of DLS. J. F. and C. H. isotope measurements. J. A. C. B. and D. M. G.: supervision, project administration and finding acquisition. All authors contributed to the final version – review & editing.

## Conflicts of interest

There are no conflicts to declare.

## Supplementary Material

SC-017-D5SC06873H-s001

## Data Availability

The data supporting this article have been included as part of the supplementary information (SI). Supplementary information: materials, experimental procedures, characterizations, SI Fig. S1–S25. See DOI: https://doi.org/10.1039/d5sc06873h.
