# Supplementary material for: Impacting the balance between CO2 and proton reduction by control over aggregation in a model π-conjugated N-heterocycle – proflavine
Source: Chem Sci. 2026 May 15;17(27):13378–86. doi: 10.1039/d5sc06873h (PMC13220174; doi:10.1039/d5sc06873h)
Supplement: SC-017-D5SC06873H-s001 [file SC-017-D5SC06873H-s001.pdf]

Supplementary Information (SI) for Chemical Science.
This journal is © The Royal Society of Chemistry 2026

Supporting Information

for

"Impacting the Balance between CO_2 and Proton Reduction by Control over Aggregation in a Model π-Conjugated N-Heterocycle – Proflavine"

Yana Reva,[‡1] Jonas Färber,[‡1] Yifan Bo,[1] Max Thiele,[1] Christian Hanke,[2] Ayşe Günay-Gürer,[1] Maximillian Herm,[1] Johannes A.C. Barth,[2] Dirk M. Guldi*[1]

Experimental Section

Materials:

Proflavine monohydrochloride (Sigma-Aldrich, >99%), triethanolamine (Sigma-Aldrich, > 99%), potassium dihydrogen phosphate (Sigma-Aldrich), dipotassium hydrogen phosphate (Fluka, 98%), ethanol (Sigma-Aldrich, >99.9%), methanol (Sigma-Aldrich, >99.8%), N,N-Dimethylformamide (DMF) (Sigma-Aldrich, 99.9%), $(Ir[dF(CF_3)ppy]_2(dtbpy))PF_6$ (Sigma-Aldrich), carbon dioxide (Linde, >99.995%), argon (Linde, >99.999%), oxygen (Air Products, >99.5%). In order to mediate pH 2.5, citrate buffer (0.1 M) was prepared with sodium citrate dehydrate (Fluka, 99%) and citric acid monohydrate (Sigma-Aldrich, ACS reagent grade).

Photocatalytic tests

To ensure reproducible photocatalytic performance, a 3.5 mL quartz cuvette was filled with 2430 µL of TEOA/KH_2PO_4-aq. mixture with selected amount of proflavine, sealed with septum, and purged with CO_2 for 15 min with 50 mbar pressure. The cuvette was then placed in front of the light source, which was a Xe-lamp with the power of 75 mW/cm^2 coupled with AM 1.5 filter. During the measurement, the solution was constantly stirred to ensure homogeneous particle dispersion.

Gas phase analysis

Generated gases were quantified through a gas chromatography (GC) setup (Shimadzu GC-2010 Plus) with packed column ShinCarbon ST 80/100 (von Restek) connected with a flame ionization detector by injecting the gas from the cuvette headspace into the GC. After set photocatalalytic

time, the amount of produced gases was measured using a gas chromatograph (GC). For every set of conditions N = 4 coherent measurements were conducted to reveal standard deviation represented by error bars.

Liquid phase analysis

CO_2 reduction products potentially generated in the liquid phase (such as formaldehyde, formic acid or methanol) were evaluated through a gas chromatography (GC) setup (Shimadzu GC-2010 Plus) with packed column CP-Sil PONA CB (50 m, 0.21 mm, 0.5 μm) P/N:CP7531 (von Agilent J&W GC Columns) connected with a thermal conductivity detector by injecting the fluid from the cuvette into the GC.

Characterization

Femtosecond transient absorption spectroscopy (fs-TAS) was performed using an Astrella-F-1K amplified Ti:sapphire femtosecond laser system from Coherent, operating at a repetition rate 1 kHz, 5.5 W power (5 mJ pulse energy), pulse duration of 80 fs. To acquire the time resolved transient absorption spectra on a sub-ps and ns resolution, an Ultrafast Systems HELIOS fs transient transient absorption spectrometer was used with time delays from 0 to 7200 ps. To acquire the time resolved transient absorption spectra on a μs resolution, an Ultrafast Systems EOS ns transient absorption spectrometer was used with time delays from 7.2 ns to 350 μs. For sub-ps, white light for the probing pulse in the visible region of the optical spectrum (~420–770 nm) was generated by focusing part of the fundamental 800 nm output onto a 2 mm sapphire disk. The excitation wavelength was generated via a TOPAS Prime from Light Conversion with standard NirUVis extension. Obtained data were treated by SVD, global and target analyses using the R-package TIMP.[1]

All the steady-state and time-resolved spectroscopic measurements have been performed at room temperature. UV-vis measurements were carried out on a Perkin Elmer Lambda 2 spectrometer or a Perkin Elmer Lambda 35 spectrophotometer. Fluoromax 3 spectrometer from Horiba Jobin Yvon was used for fluorescence/3D fluorescence measurements in the visible region.

Dynamic Light Scattering (DLS) analysis was conducted with Zetasizer Nano ZS from Malvern Instruments GmbH with green laser (532 nm). The solution was buffered with 4.28 vol% TEOA and 0.55 M KH_2PO_4. For every concentration N = 3 coherent measurements were conducted to reveal standard deviation represented by error bars.

Raman spectra were acquired with a WiTec alpha 300r confocal Raman microscope. A HeNe laser with an output of 633 nm was used for sample excitation. To perform the operando experiments in electrolyte, a in a home-built Teflon cell with three electrodes was utilized and controlled by Methrom µAutlobab FraIII, wheras the lens on the microscope was focused on the working electrode above the cell (no immersion). The cell was equipped with a reference electrode (Ag/AgCl), a counter electrode (Pt ring), and a working electrode with the catalyst drop-casted on a platinum. Electrolyte was purged with CO_2 prior to experiment. After application of a potential of -1.45 V for 30 seconds, Raman spectrum was gathered as fast as possible to avoid proflavine desorption. Longer measurements or time-dependent measurements are limited by the fast degradation of proflavine under laser and partial water reduction.

Mass spectrometry measurements were performed with TSQ Quantum Access MAX, Thermo Scientific with an electrospray ionization (ESI) source. The sample solutions were directly injected into the ESI source via a syringe pump at a flow rate of 2.5-3.5 µl/min. For the negative ion mode the capillary voltage was set to + 4.5 kV and the end plate offset to + 0.5 kV.

Laser spectroscopy for isotope ratios of CO_2 and CH_4

Samples were analyzed for their $^{13}C/^{12}C$ ratios by wavelength-scanned cavity ring-down spectroscopy (Picarro G2201-i). Here samples were admitted via an autosampler to the instrument via 12-mL-Exetainer(R) vials with butyl caps with regular admission of control, standard and blank samples. Isotope ratios are expressed as

$$\delta^{13}C\ (‰) = ((R_{sample} / R_{standard}) - 1) * 1000$$

with R being the $^{13}C/^{12}C$ ratios and the standard Vienna Pee Dee Belemnite with a defined isotope ratio of 0.0111802. [2] One-σ standard deviations of measurements were better than ± 0.2 for ‰ for $\delta^{13}C_{CO2}$ and $\delta^{13}C_{CH4}$. Note that when CO_2 was measured together with all the other organic chemicals (Pf, TEOA **and** buffer), interferences likely caused larger deviations of up to 5.8 ‰.

Nonetheless repetition was better at higher CO_2 concentrations and average $\delta^{13}C_{CO2}$ values are still valid.

Isotope ratio mass spectrometry for isotope ratios of dissolved organic carbon

Stable isotope ratios and concentrations of DOC were determined using persulfate wet oxidation on a TIC-TOC analyzer (Aurora 1030 W, OI Analytical, College Station, Texas, USA). This pre-analyzer was coupled to an isotope ratio mass spectrometer (IRMS, Delta V plus, ThermoFisher, Bremen, Germany). Details of the analytical procedure are available in St-Jean (2003) and van Geldern et al. (2013). Isotope ratios were expressed in the d-notation and one-σ standard deviations of repeat $\delta^{13}C_{doc}$ were better than ±0.3 ‰.[3, 4]

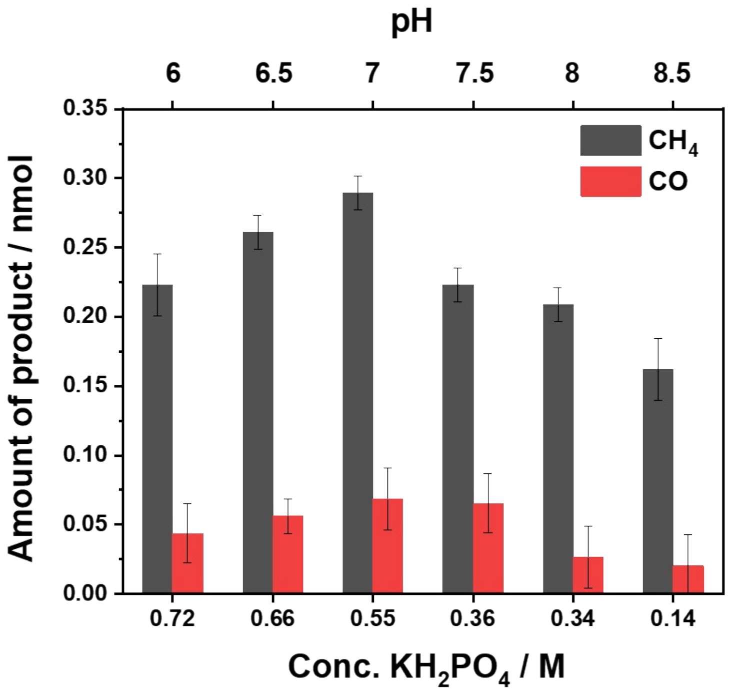

Figure S1. pH-dependent photocatalytic CH_4/CO generation efficiency (pH is altered by means of KH_2PO_4 concentration variation). The cuvette was filled with aqueous 4.28 vol% TEOA and 17.7 μM **(Pf-H)**$^+$, whereas the intensity of the irradiating light was set at 75 mW/cm^2 in the presence of an AM 1.5 filter.

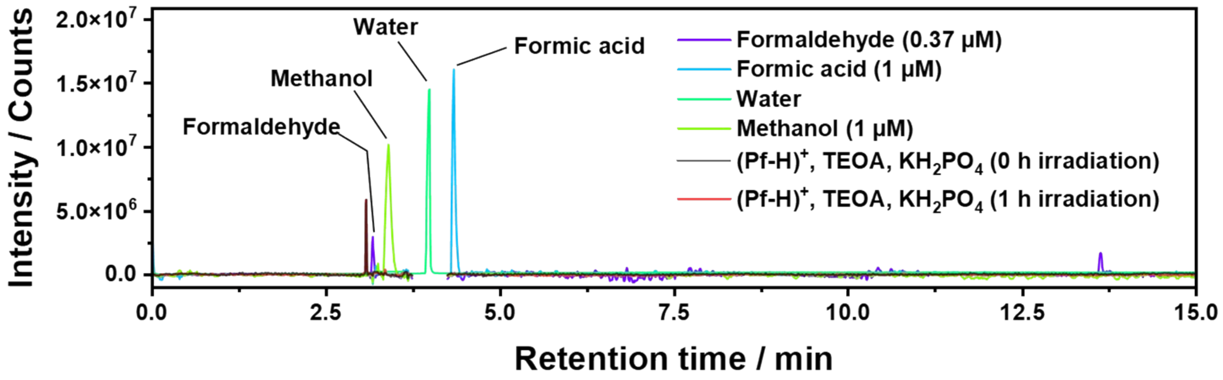

Figure S2. GC analysis of the liquid phase under optimized photocatalytic conditions (25 μM **(Pf-H)**$^+$ in aqueous solutions with 4.28 vol% TEOA and 0.55 M KH_2PO_4) before (black) and after 1 of irradiation (red). Notably, no formaldehyde, methanol nor formic acid were detected.

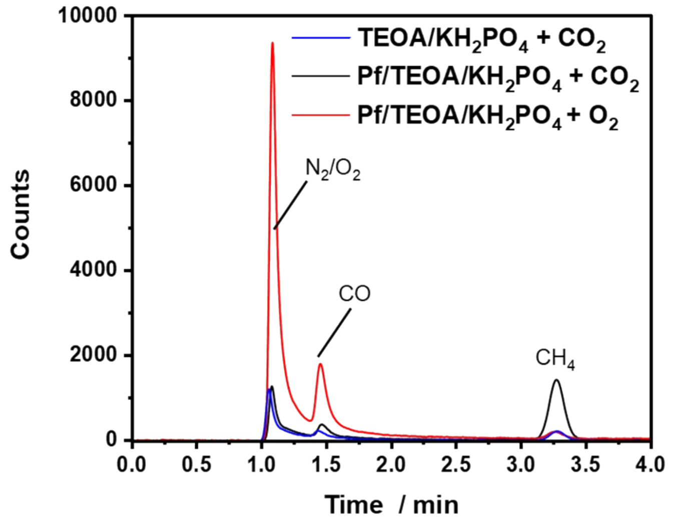

Figure S3. GC analysis (FID) of the gases, generated within a photocatlatic system with 25 μM **(Pf-H)**$^+$, 4.28 vol% TEOA and 0.55 M KH_2PO_4 at a pH of 7, comparing CO_2 vs. O_2-saturated system.

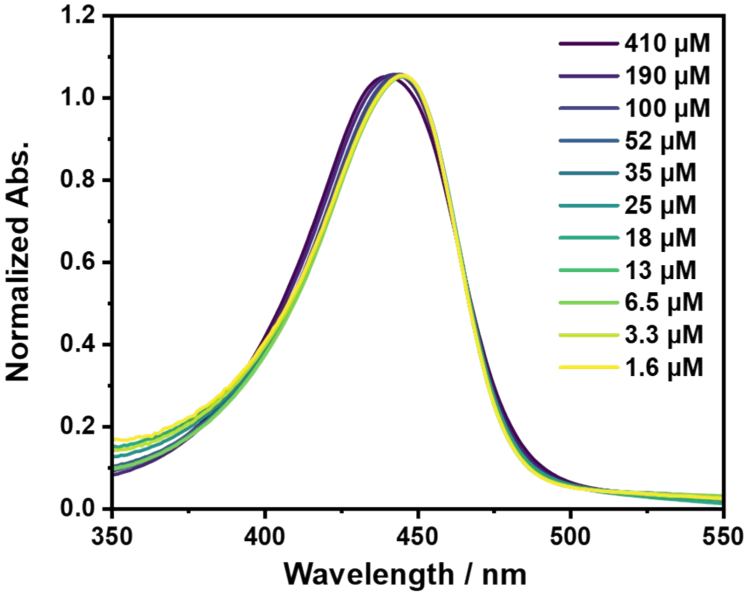

Figure S4. Normalized absorption spectra of **(Pf-H)**$^{+}$ at various concentrations.

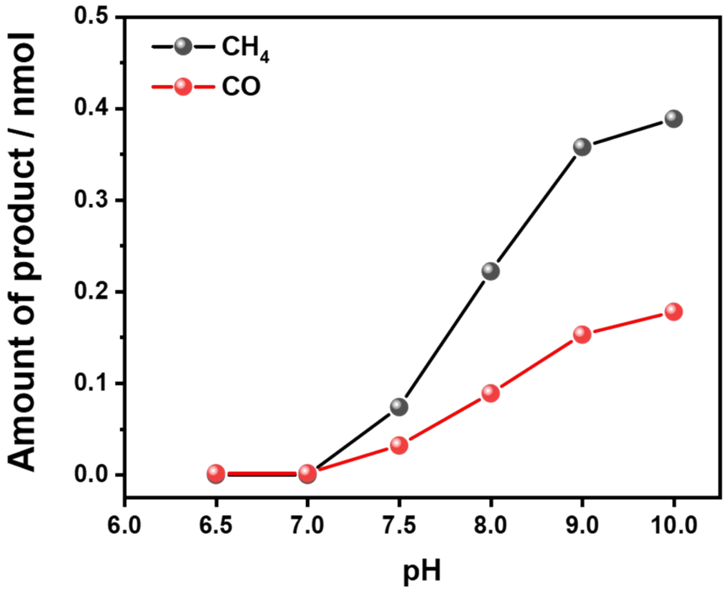

Figure S5. pH-dependent photocatalytic CH_4 and CO generation efficiency (pH is altered by means of KH_2PO_4 concentration variation). The cuvette was filled with aqueous 4.28 vol% TEOA and 1000 μM **(Pf-H)**$^+$, whereas the intensity of the irradiating light was set at 75 mW/cm^2 in the presence of an AM 1.5 filter.

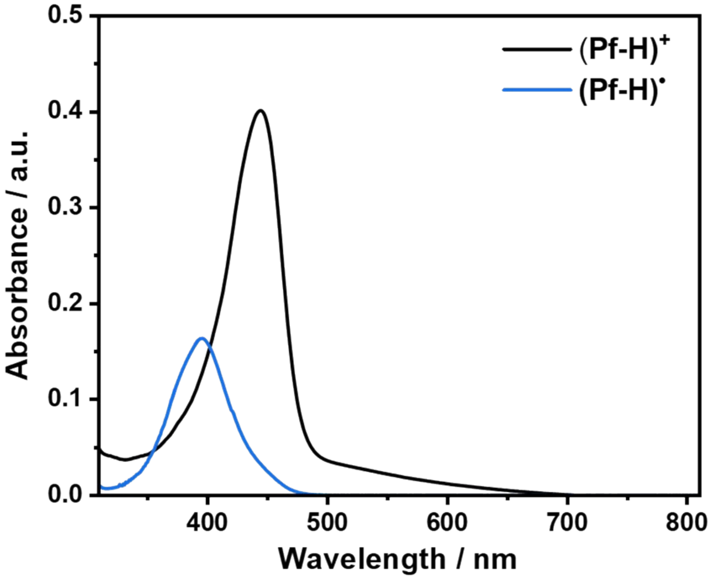

Figure S6. Absorption spectra of **(Pf-H)**$^+$ in aqueous solutions before (black) and after chemical reduction with $LiAlH_4$ (blue).

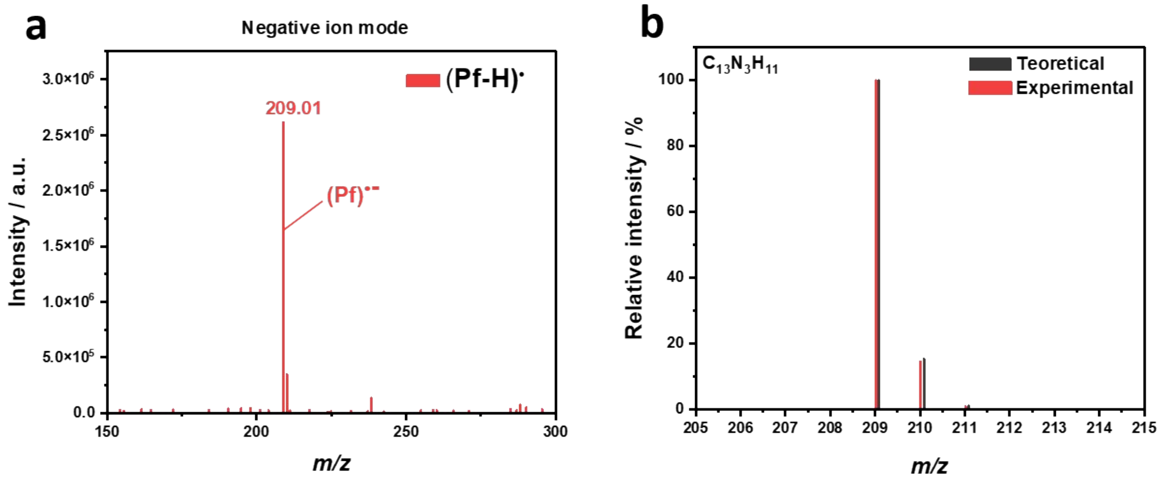

Figure S7. **a**, ESI-MS spectrum of **(Pf-H)•** measured in the negative ion mode. **b**, Comparison of the experimental data with the corresponding isotopic pattern.

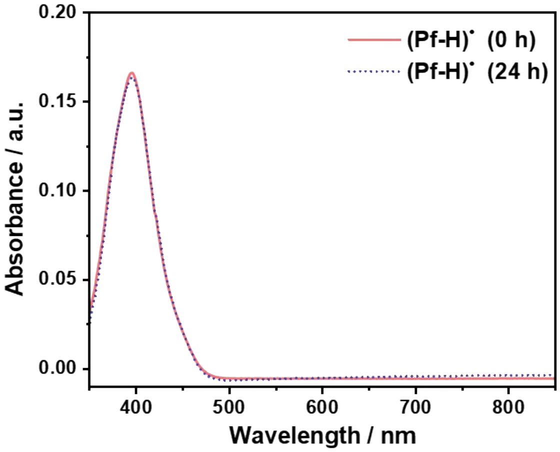

Figure S8. Absorption spectra of **(Pf-H)$^{\bullet}$** in oxygen-free aqueous solution with different time delays.

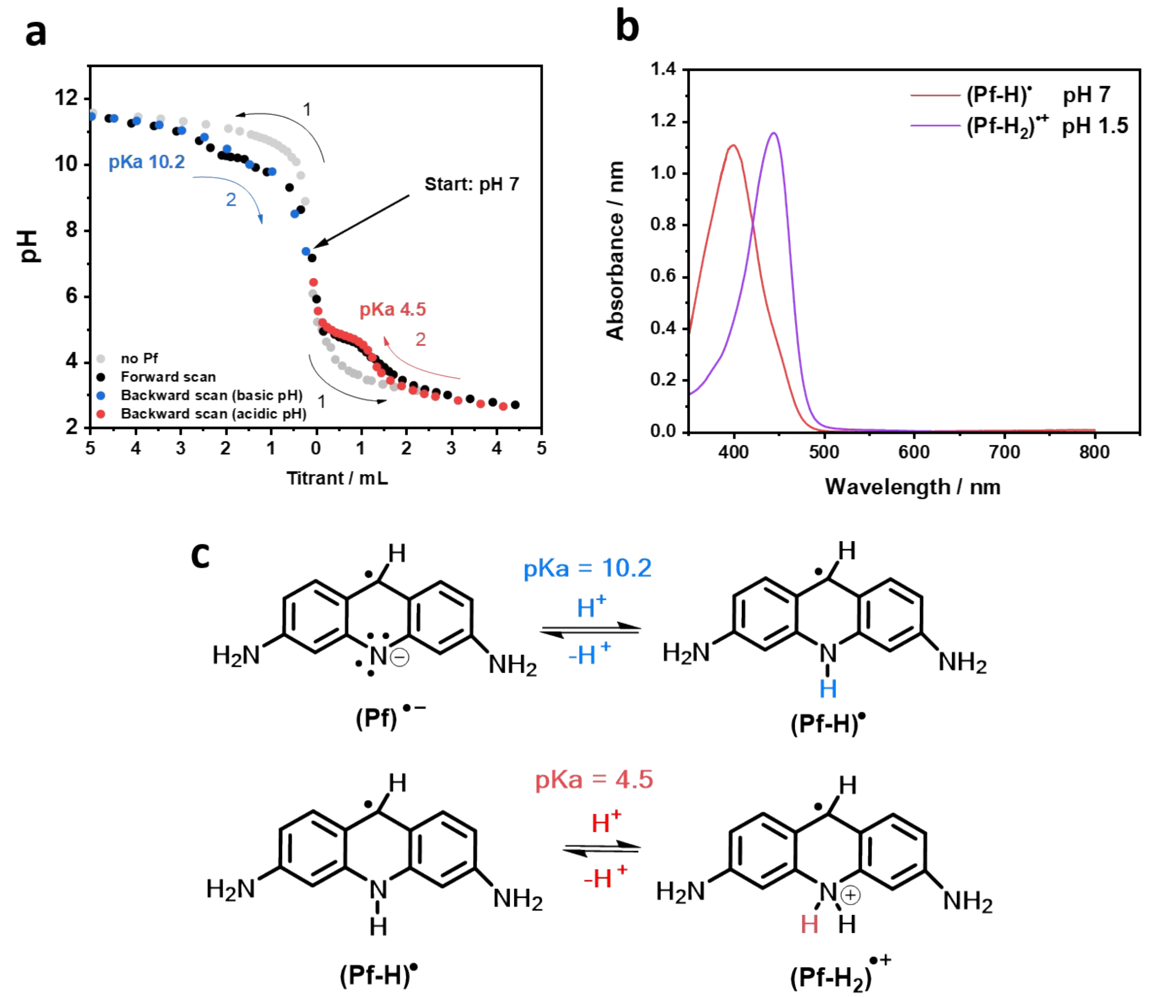

Figure S9. a, Reversible titration curves of chemically reduced **(Pf-H)**$^{\bullet}$. For acidic conditions HCl (0.1 M) was used as titrant and for basic sweeps NaOH (0.1 M). **b**, Absorption spectra of **(Pf-H)**$^{\bullet}$ and **(Pf-H$_2$)**$^{\bullet+}$. **c**, Corresponding structures and pKa related transformations.

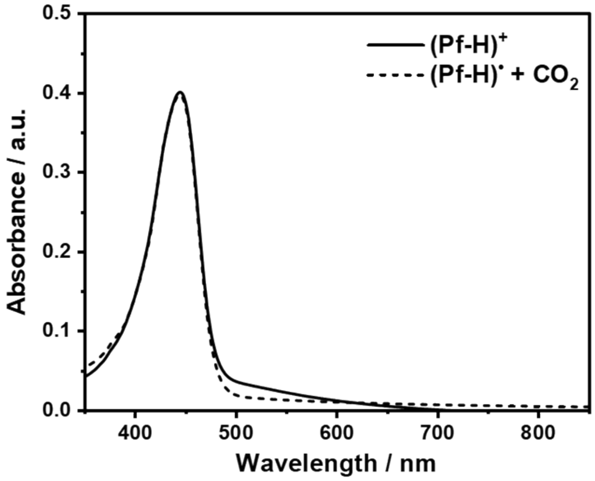

Figure S10. Absorption spectra of (**Pf-H**)$^{\bullet}$ after re-exposure to CO_2 and compared with (**Pf-H**)$^{+}$.

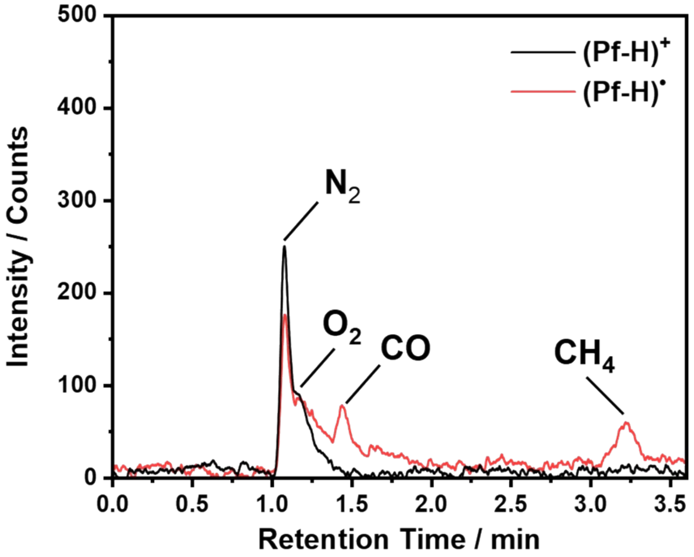

Figure S11. GC analysis of the generated gases upon exposure of **(Pf-H)**$^+$ (black) and **(Pf-H)**$^{\bullet}$ (red) to CO_2.

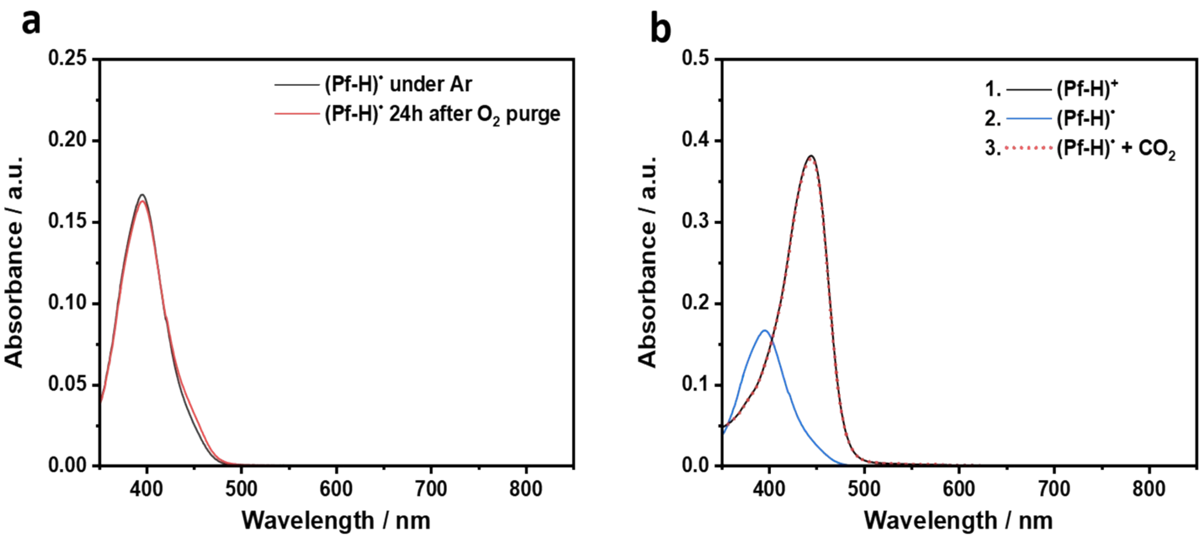

Figure S12 a, Change of **(Pf-H)˙** absorption after 24 hours in O_2-enreached aqueous solution (only 2% of **(Pf-H)˙** was re-oxidized). **b,** Absorption spectra of **(Pf-H)**$^+$ before reduction and after re-oxidation from its reduced state.

Table S1. Isotopic mass spectrometry measurements of various reference measurements, with variations of the inclusion of key components in the photocatalytic systems. The complete photocatalytic system was measured with three different CO_2 amounts, where either 100, 200 or 300 µL of 100% CO_2 were added.

Pf	TEOA	Buffer	CO_2	Irradiation	$\delta^{13}C_{CO2}$ VPDB [‰]	c(CO_2) [ppm]	$\delta^{13}C_{CH4}$ VPDB [‰]	c(CH_4) [ppm]
	x	x	x	x	-32.9	1020	-	2.6
x		x	x	x	-30,.7	1600	-	2.5
x	x	x	x		-26.5	1030	-	3.1
			x		-41.4	4400	-	<1
			x		-41.5	4400	-	<1
			x		-41.7	3900	-	<1
x	x	x			-18.9	103	-	<1
x				x	-39.7	3920	-	<1
x	x	x		x	-	165	-	<1
x		x		x	-	223	-	<1
	x	x		x	-	196	-	<1
x	x	x	x	x (100 µL)	-48.7	600	-37.8	660
x	x	x	x	x (100 µL)	-44.4	710	-37.8	610
x	x	x	x	x (100 µL)	-49.4	640	-37.8	710
x	x	x	x	x (200 µL)	-49.1	1100	-37.2	670
x	x	x	x	x (200 µL)	-48.8	1100	-37.3	730
x	x	x	x	x (200 µL)	-43.3	1100	-37.2	710
x	x	x	x	x (300 µL)	-48.5	1800	-36.9	730
x	x	x	x	x (300 µL)	-48.6	1800	-36.8	760
x	x	x	x	x (300 µL)	-48.8	1800	-36.8	750

Table S2. Isotope mass spectrometry measurements of the dissolved organic carbon (doc) of the photocatalytic system with and without irradiation.

Name	$\delta^{13}C_{doc}$ VPDB [‰]	c (doc) [ppm]
Not irradiated sample	-31.0	45700
Irradiated sample	-31.2	41800

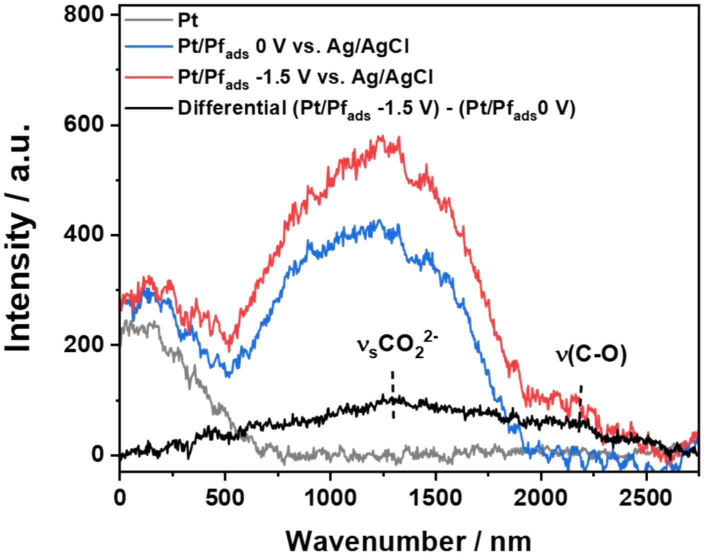

Figure S13. Raman spectroscopy applied to electrochemical CO_2 reduction of Proflavine on Pt in CO_2-purged water with 0.1 M KH_2PO_4 and K_2HPO_4 (pH 7).

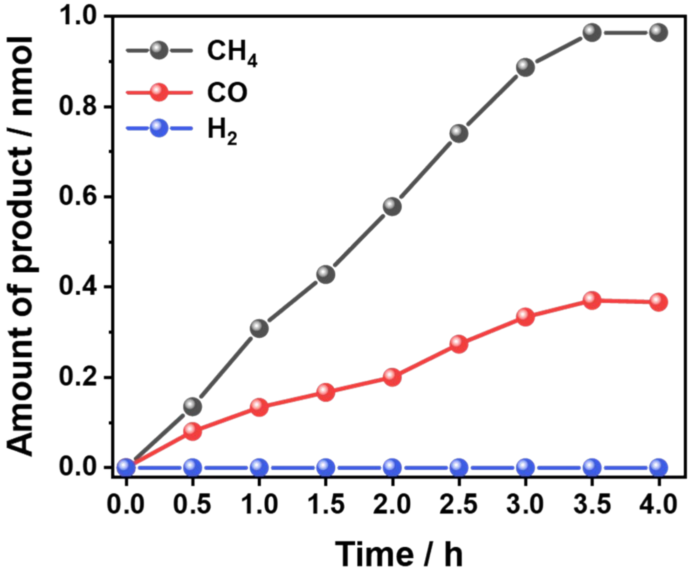

Figure S14. Cumulative rate of CH_4, CO, and H_2 evolution with 25 μM **(Pf-H)**$^+$ in water with 4.28 vol% TEOA and 0.55 M KH_2PO_4 upon 1 sun irradiation with a Xe-Lamp equipped with 1.5 AM filter measured over total of 4 hours.

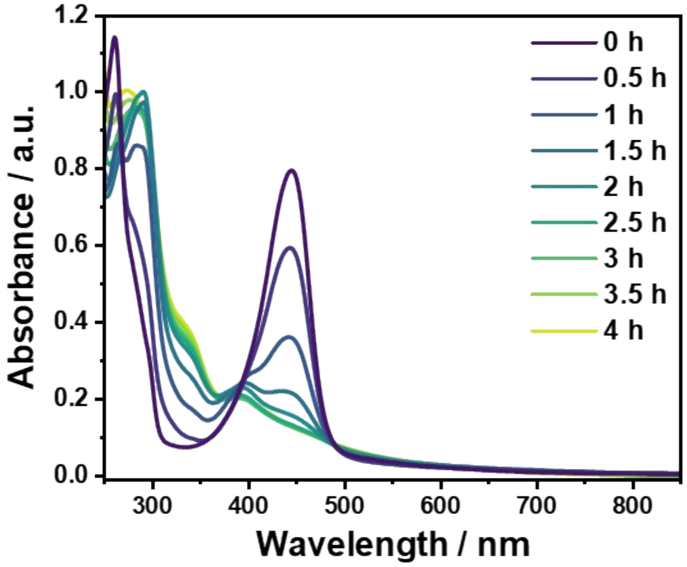

Figure S15. Absorption spectra of 25µmol **(Pf-H)**$^+$ taken every 30 min during photocatalysis in water with 4.28 vol% TEOA and 0.55 M KH_2PO_4 upon 1 sun irradiation with a Xe-Lamp equipped with 1.5 AM filter.

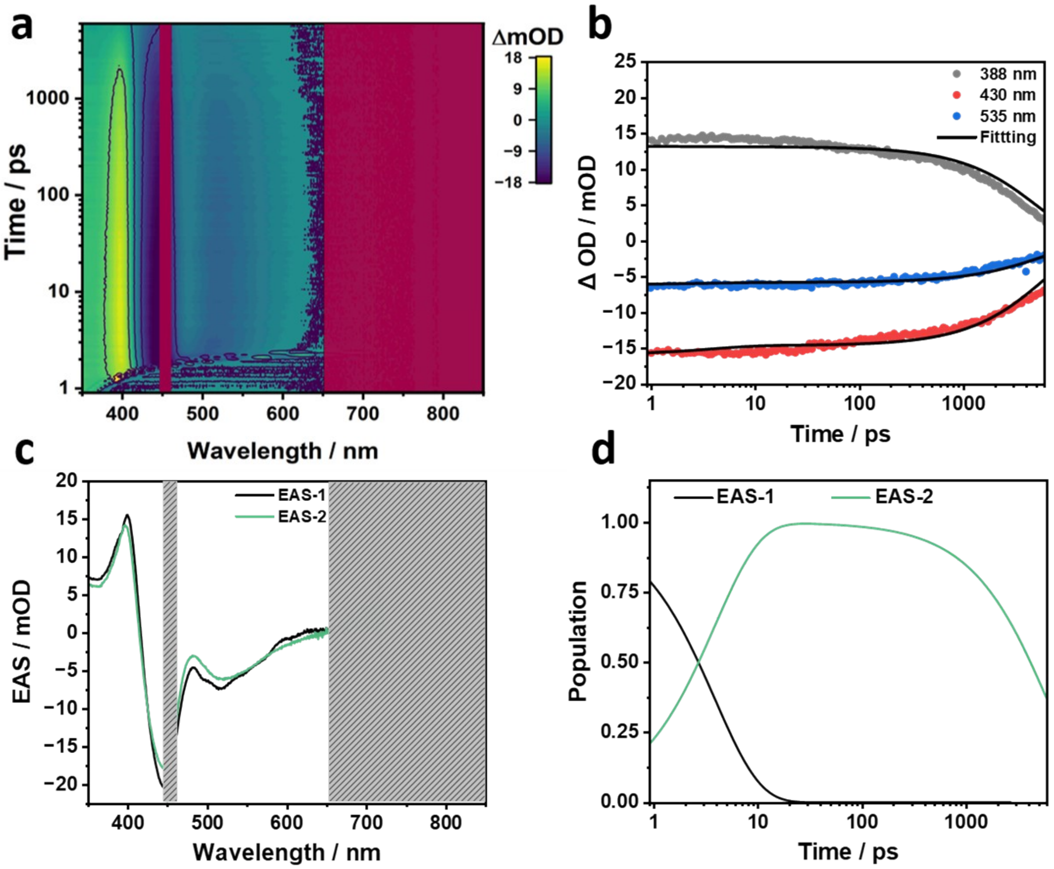

Figure S16. a, fs-TA heat map of 25 μM **(Pf-H)**$^+$ in aqueous solutions with 0.55 M K_2HPO_4/KH_2PO_4 and purged with CO_2. The photoexcitation wavelength was set to 450 nm. **b**, Representative time traces at 388, 430, and 535 nm depicting the recorded evolution of transients. **c**, Evolution associated spectra (EAS) from sequential two-exponential global analysis of the fs-TA spectra: EAS-1 and EAS-2 correspond to (S_1^{hot})**(Pf-H)**$^+$ with a lifetime of 3.8 ps and (S_1^{rel})**(Pf-H)**$^+$ with a lifetime of 4.7 ns, respectively. **d**, Time profiles depicting the time-resolved population of the corresponding EASs from global analyses of the fs-TA spectra.

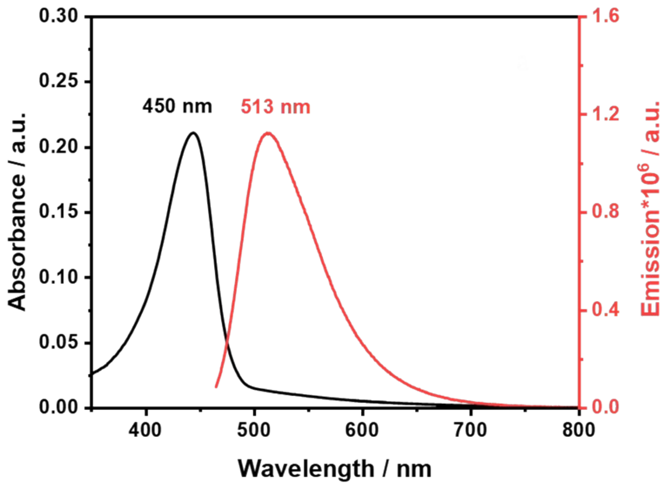

Figure S17. Absorption (black) and emission spectra (red) of 25 μM **(Pf-H)**$^+$ in aqueous solutions with KH_2PO_4/K_2HPO_4 at pH 7. The photoexcitation wavelength was set to 450 nm.

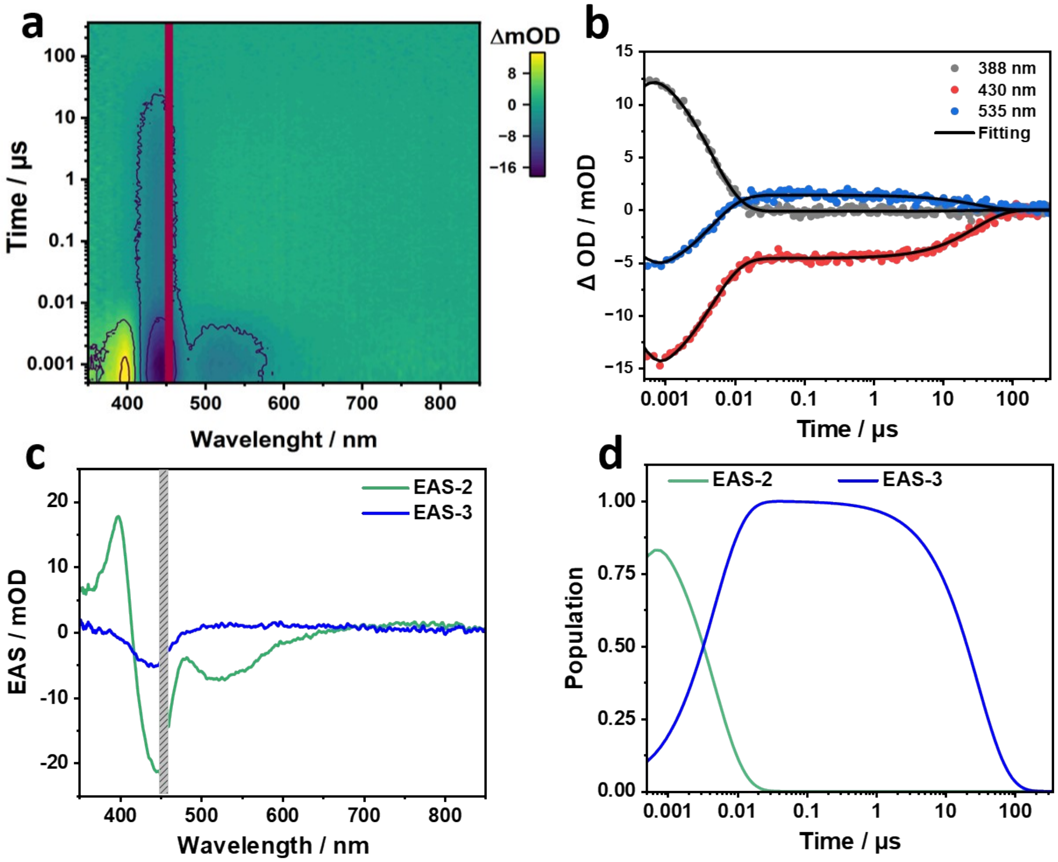

Figure S18. **a**, ns-TA heat map of 25 µM **(Pf-H)**$^+$ in aqueous solutions with 0.55 M K_2HPO_4/KH_2PO_4 and purged with CO_2. The photoexcitation wavelength was set to 450 nm. **b**, Representative time traces at 388, 430, and 535 nm depicting the recorded evolution of transients. **c**, Evolution associated spectra (EAS) from sequential two-exponential global analyses of the ns-TA spectra: (S_1^{rel})**(Pf-H)**$^+$ and (T_1)**(Pf-H)**$^+$ with lifetimes of 4.7 ns and 29.2 µs, respectively. **d**, Time profiles depicting the time-resolved population of the corresponding EASs from global analyses of the ns-TA spectra.

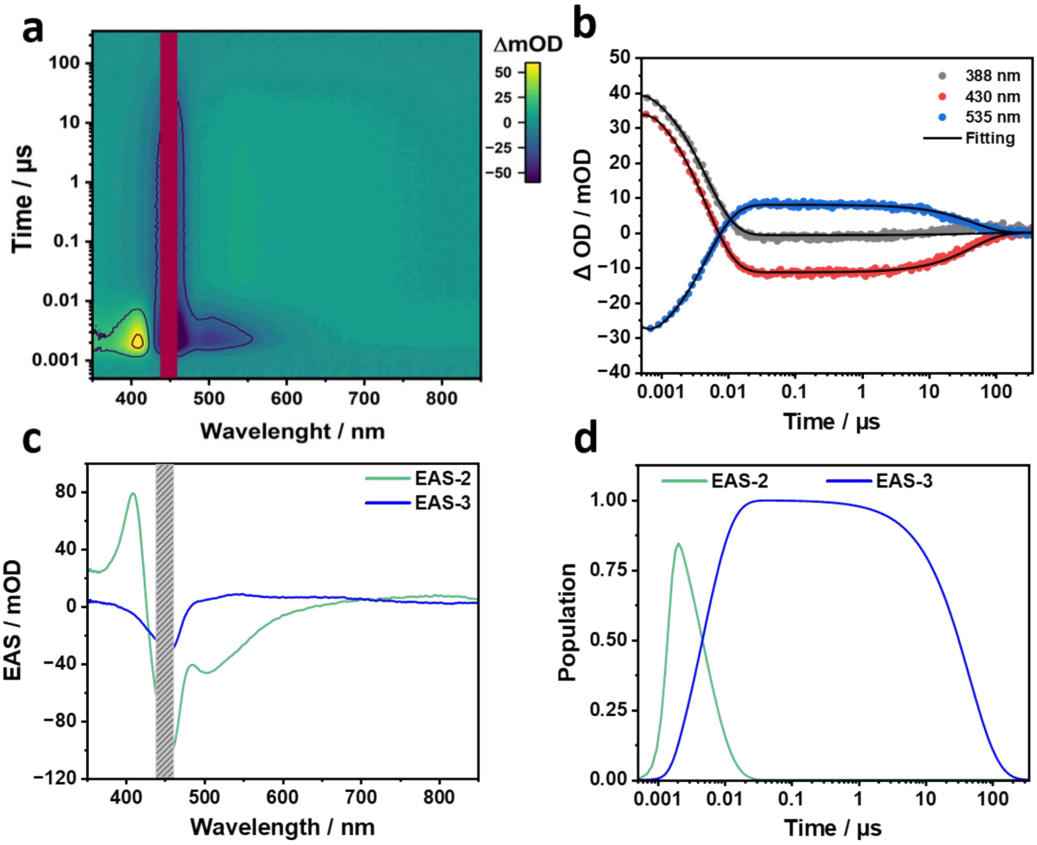

Figure S19. **a**, ns-TA heat map of 25 µM **(Pf-H)**$^+$ in ethanol and purged with argon. The photoexcitation wavelength was set to 450 nm. **b**, Representative time traces at 388, 430, and 535 nm depicting the recorded evolution of transients. **c**, Evolution associated spectra (EAS) from sequential two-exponential global analyses of the ns-TA spectra: (S_1^{rel})**(Pf-H)**$^+$ and (T_1)**(Pf-H)**$^+$ with lifetimes of 4.7 ns and 44.8 µs, respectively. **d**, Time profiles depicting the time-resolved population of the corresponding EASs from global analyses of the ns-TA spectra.

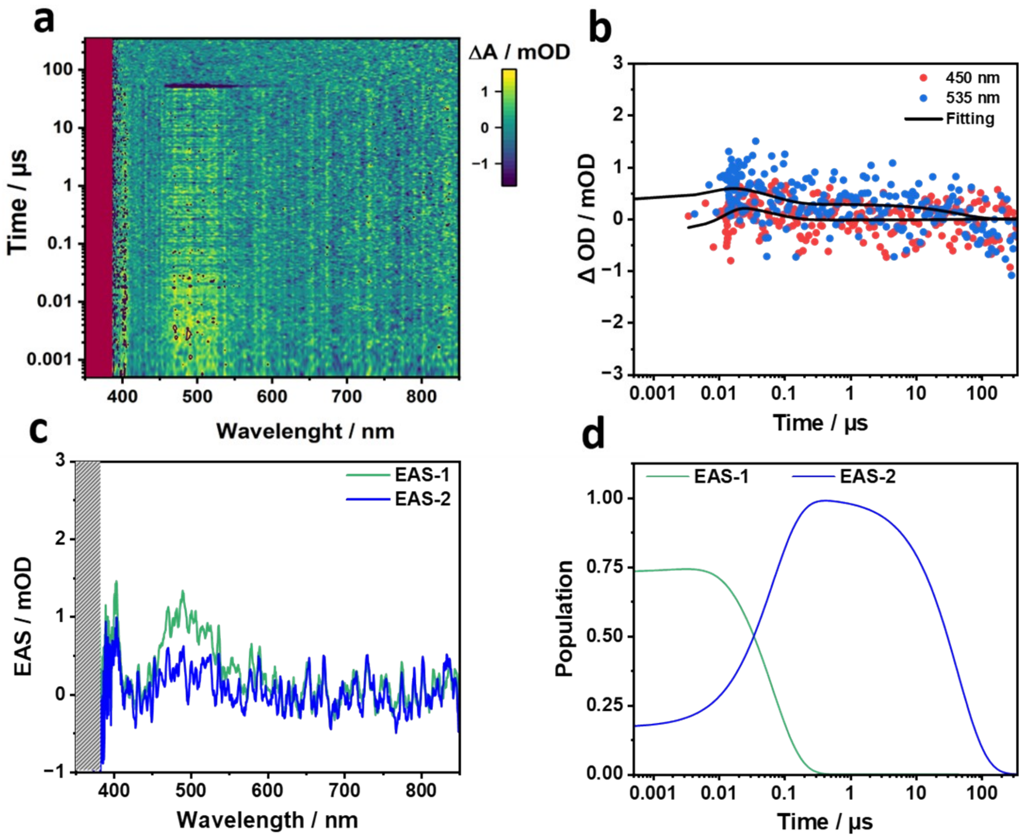

Figure S20. **a**, ns-TA heat map of 100 µM $(Ir[dF(CF_3)ppy]_2(dtbpy))PF_6$ in ethanol and purged with argon. The photoexcitation wavelength was set to 360 nm. **b**, Representative time traces at 450 and 535 nm depicting the recorded evolution of the transients. **c**, Evolution associated spectra (EAS) from sequential two-exponential global analyses of the ns-TA spectra: EAS-1, exhibiting a lifetime of 66 ns, is followed by EAS-2, which corresponds to the fully formed (T_1) with a lifetime of 43 µs. **d**, Time profiles depicting the time-resolved population of the corresponding EASs from global analyses of the ns-TA spectra.

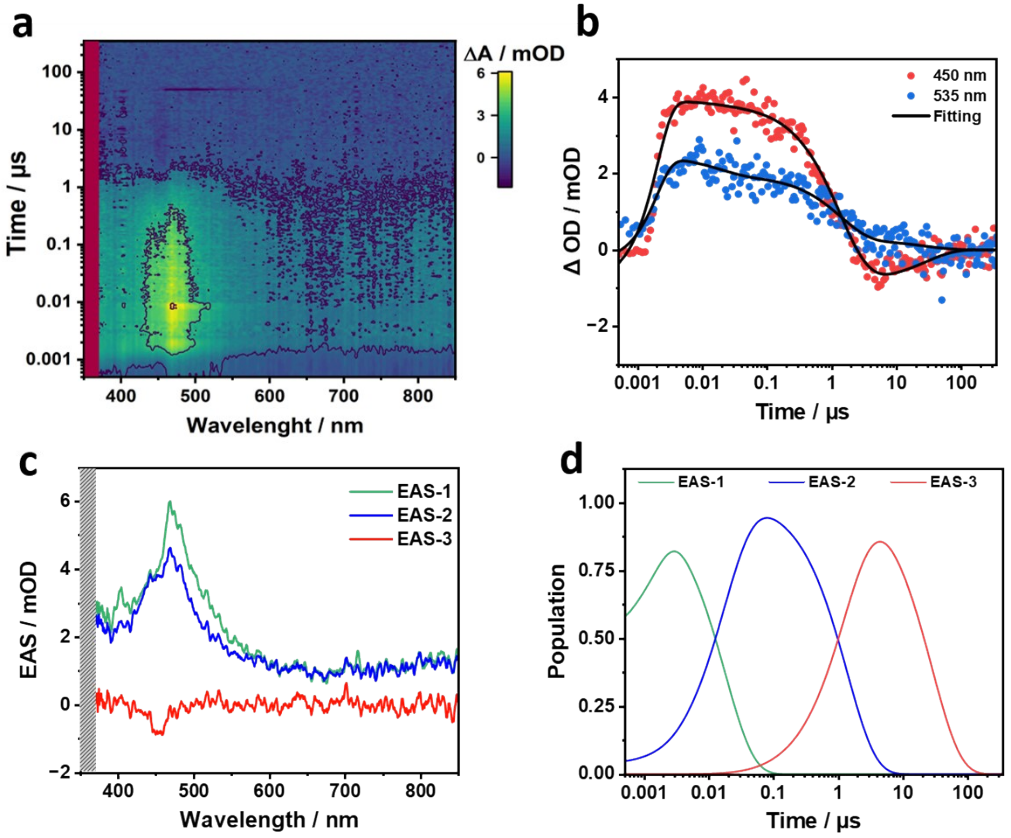

Figure S21. a, ns-TA heat map of 100 µM (Ir[dF(CF_3)ppy]$_2$(dtbpy))PF_6 and 250 µM **(Pf-H)**$^+$ in ethanol and purged with argon. The photoexcitation wavelength was set to 360 nm. **b**, Representative time traces at 450 and 535 nm depicting the recorded evolution of the transients. **c**, Evolution associated spectra (EAS) from sequential three-exponential global analyses of the ns-TA spectra: EAS-1 with a lifetime of 18 ns, EAS-2 corresponds to (T_1) of (Ir[dF(CF_3)ppy]$_2$(dtbpy))PF_6 with a 1.38 µs lifetime, and EAS-3 corresponds to (T_1) of **(Pf-H)**$^+$ with a 28.2 µs lifetime. **d**, Time profiles depicting the time-resolved population of the corresponding EASs from global analyses of the ns-TA spectra.

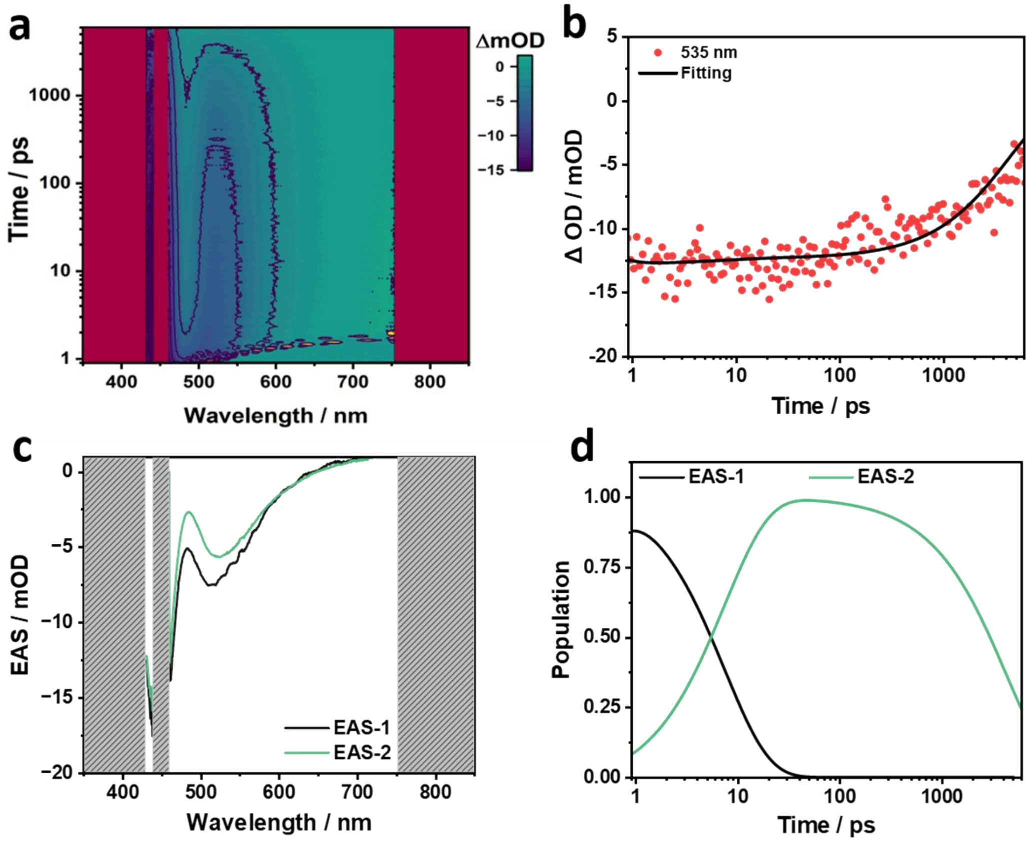

Figure S22. a, fs-TA heat map of 25 μM **(Pf-H)**$^+$ in aqueous solutions with 4.28 vol% TEOA, 0.55 M KH_2PO_4, and purged with CO_2. The photoexcitation wavelength was set to 450 nm. **b**, Representative time trace at 535 nm depicting the recorded evolution of the transient. **c**, Evolution associated spectra (EAS) from sequential two-exponential global analyses of the fs-TA spectra: EAS-1 and EAS-2 correspond to (S_1^{hot})**(Pf-H)**$^+$ with a lifetime of 7.5 ps and (S_1^{rel})**(Pf-H)**$^+$with a lifetime of 4.5 ns, respectively. **d**, Time profiles depicting the time-resolved population of the corresponding EASs from global analyses of the fs-TA spectra.

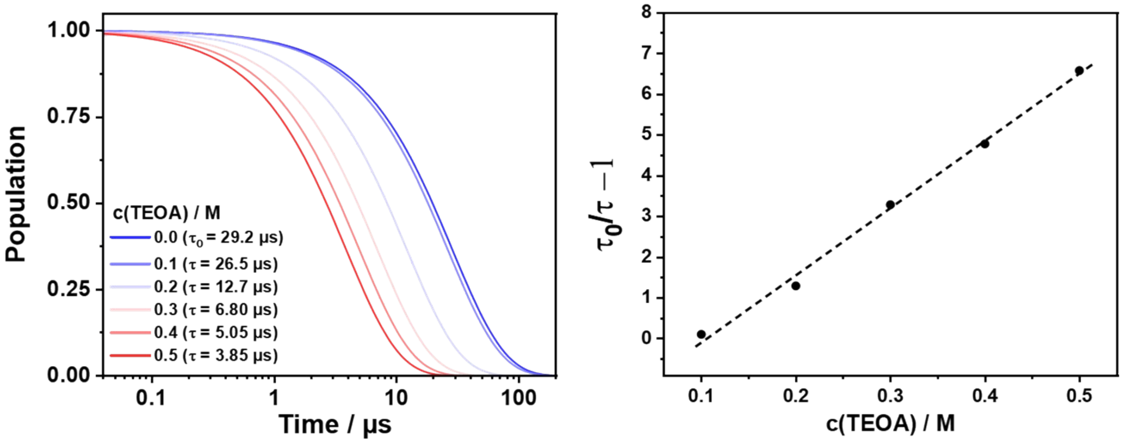

Figure S23. a, Population of (T_1)**(Pf-H)**$^+$ at different TEOA concentrations and pH 7. **b,** Stern−Volmer plot derived from ns-TA spectra with a slope of 16.4 M^{-1} (k_{SV}).

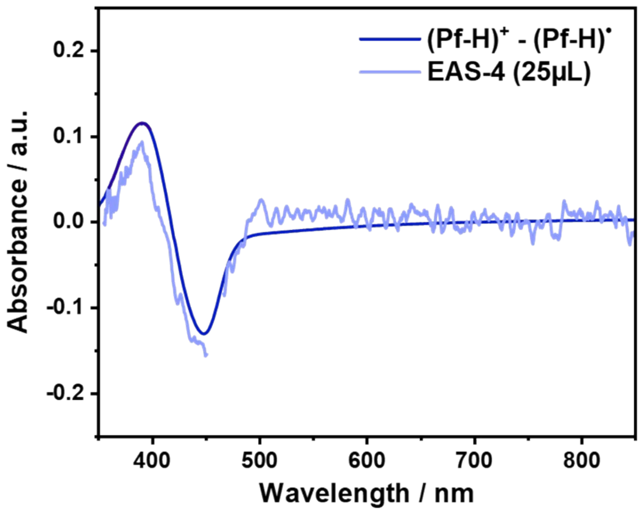

Figure S24. Comparison of the **(Pf-H)**$^+$ – (**Pf-H)**$^•$ with the EAS-4 in ns-TA of **(Pf-H)**$^+$ (25 μM) dissolved in aqueous solution with 4.28 vol% TEOA and 0.55 M KH_2PO_4.

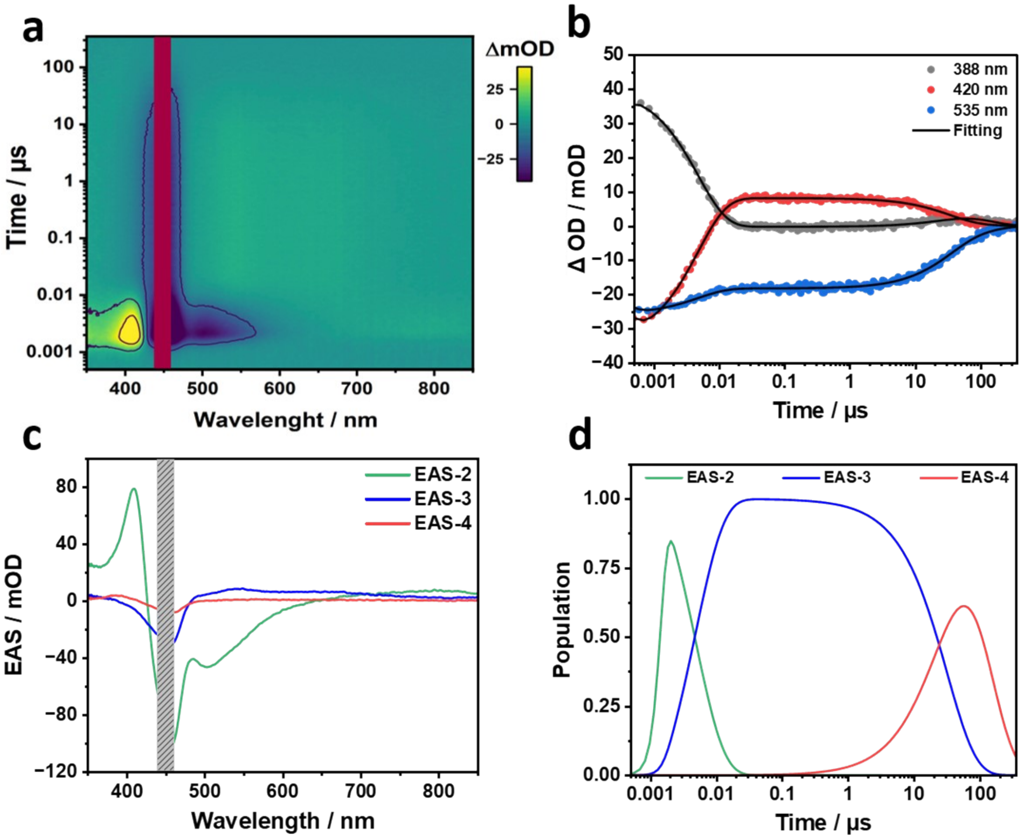

Figure S25. a, ns-TA heat map of 25 µM **(Pf-H)**$^+$ in water with 4.28 vol% methanol and purged with CO_2. The photoexcitation wavelength was set to 450 nm. **b**, Representative time traces at 388, 430, and 535 nm depicting the recorded evolution of the transients. **c**, Evolution associated spectra (EAS) from sequential three-exponential global analyses of the ns-TA spectra: (S_1^{rel})**(Pf-H)**$^+$ and (T_1)**(Pf-H)**$^+$ with lifetimes of 4.7 ns and 31.8 µs. The last species corresponds to **(Pf-H)**$^\bullet$ with a lifetime of 115 µs. **d**, Time profiles depicting the time-resolved population of the corresponding EASs from global analyses of the ns-TA spectra.

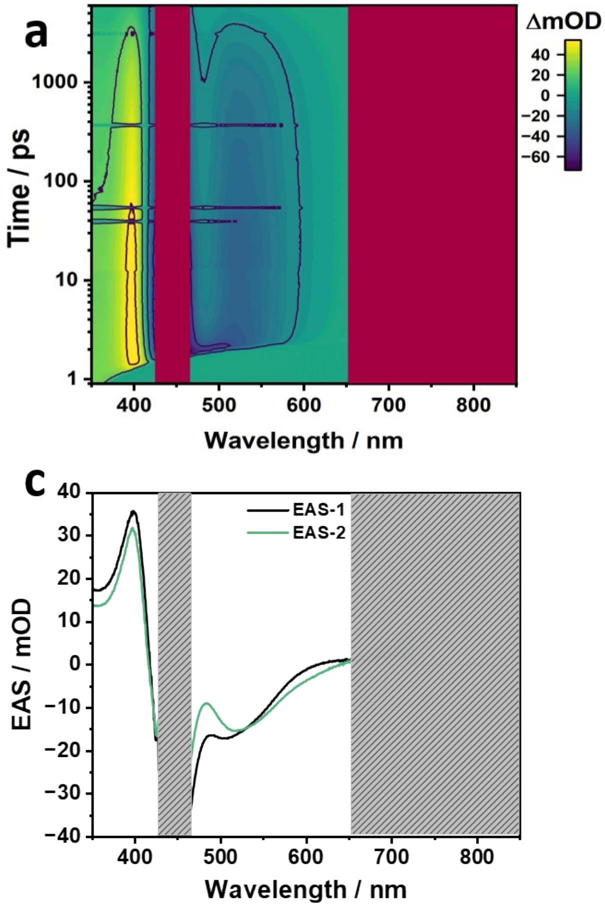

Figure S26. **a**, fs-TA heat map of 410 µM **(Pf-H)**$^+$ in aqueous solutions with 0.55 M K_2HPO_4/KH_2PO_4 and purged with CO_2. The photoexcitation wavelength was set to 450 nm. **b**, Representative time traces at 388 and 535 nm depicting the recorded evolution of the transients. **c**, Evolution associated spectra (EAS) from sequential two-exponential global analyses of the fs-TA spectra: EAS-1 and EAS-2 correspond to (S_1^{hot})**(Pf-H)**$^+$ with a lifetime of 3.2 ps and (S_1^{rel})**(Pf-H)**$^+$ with a lifetime of 5 ns, respectively. **d**, Time profiles depicting the time-resolved population of the corresponding EASs from global analyses of the fs-TA spectra.

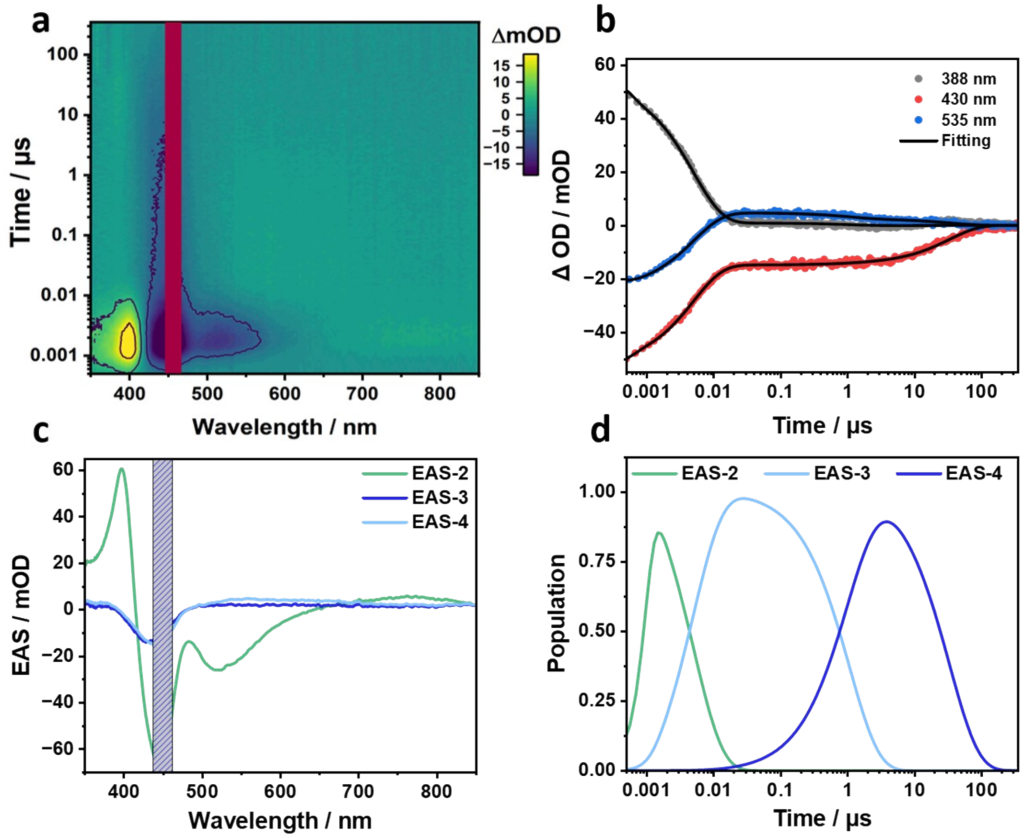

Figure S27. **a**, ns-TA heat map of 410 μM **(Pf-H)**$^{+}$ in aqueous solutions with 0.55 M K_2HPO_4/KH_2PO_4 and purged with CO_2. The photoexcitation wavelength was set to 450 nm. **b**, Representative time traces at 388, 430, and 535 nm depicting the recorded evolution of the transients. **c**, Evolution associated spectra (EAS) from sequential three-exponential global analyses of the ns-TA spectra: (S_1^{rel})**(Pf-H)**$^{+}$ with a lifetime of 5 ns, (T_1)**(Pf-H)**$^{+}$ with a lifetime of 1 μs, and (T_1)**(Pf-H)**$^{+}$ with a lifetime of 33 μs. **d**, Time profiles depicting the time-resolved population of the corresponding EASs from global analyses of the ns-TA spectra.

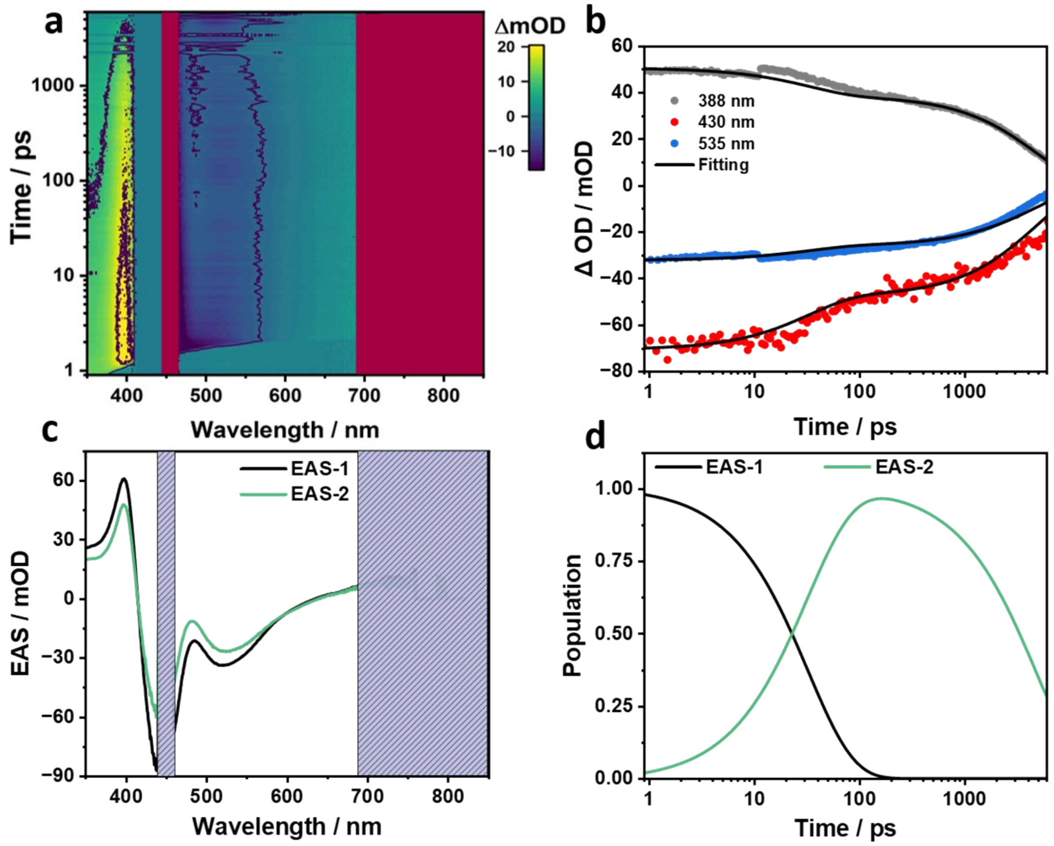

Figure S28. a, fs-TA heat map of 410 μM **(Pf-H)**$^+$ in aqueous solutions with 4.28 vol% TEOA, 0.55 M KH_2PO_4, and purged with CO_2. The photoexcitation wavelength was set to 450 nm. **b**, Representative time traces at 388, 430, and 535 nm depicting the recorded evolution of the transients. **c**, Evolution associated spectra (EAS) from sequential two-exponential global analyses of the fs-TA spectra: EAS-1 and EAS-2 correspond to (S_1^{hot})**(Pf-H)**$^+$ with a lifetime of 33.3 ps and (S_1^{rel})**(Pf-H)**$^+$ with a lifetime of 4.5 ns, respectively. **d**, Time profiles depicting the time-resolved population of the corresponding EASs from global analyses of the fs-TA spectra.

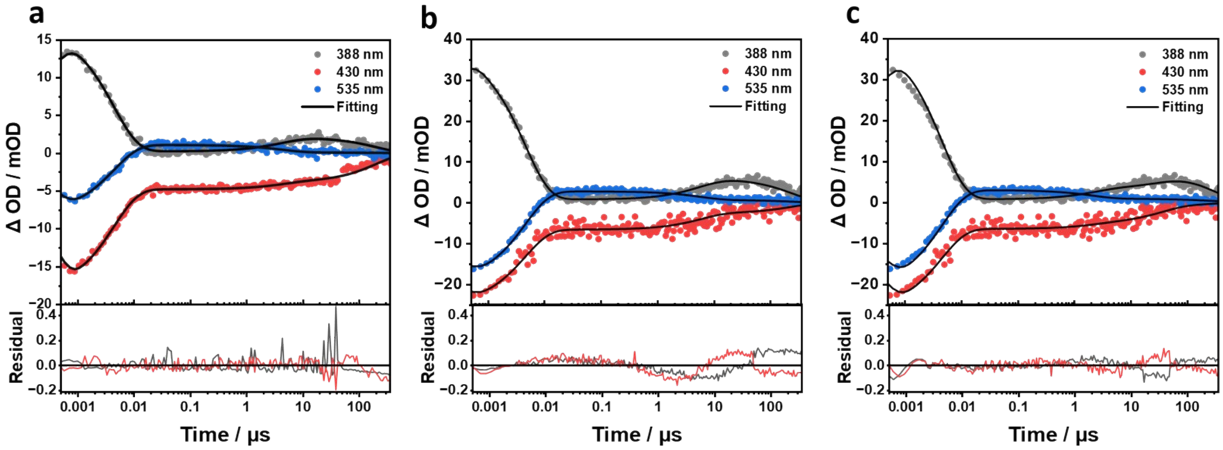

Figure S29. Time traces depicting the evolution of transients from a sequential three-exponential global analyses of the ns-TA spectra of **a**, 25 μM **(Pf-H)**$^+$ and **b**, 410 μM **(Pf-H)**$^+$ and four-exponential global analysis of **c**, 410 μM **(Pf-H)**$^+$ with the corresponding residual matrices (the first singular vector is shown in black; the second in red). Samples are measured in aqueous solutions with 4.28 vol% TEOA, 0.55 M KH_2PO_4, and purged with CO_2.

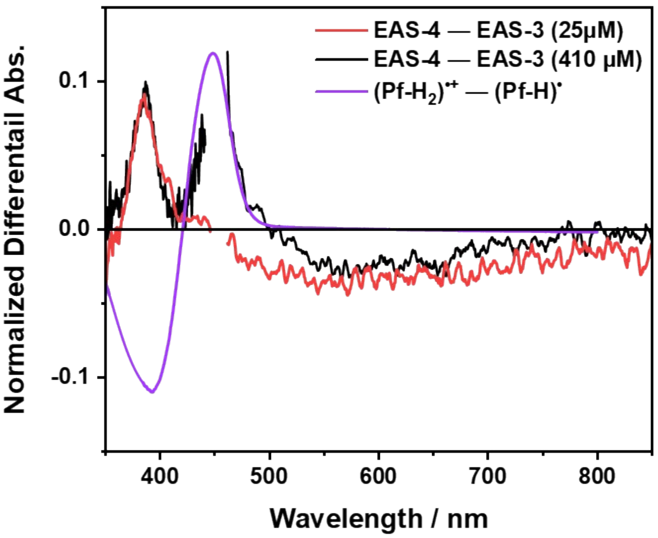

Figure S30. Comparison of the **(Pf-H$_2$)$^{•+}$** – **(Pf-H)$^{•}$** with the differential EAS-4 – EAS-3 ns-TA of 410 μM **(Pf-H)$^{+}$** and 25μM in aqueous solutions with 4.28 vol% TEOA and 0.55 M KH_2PO_4.

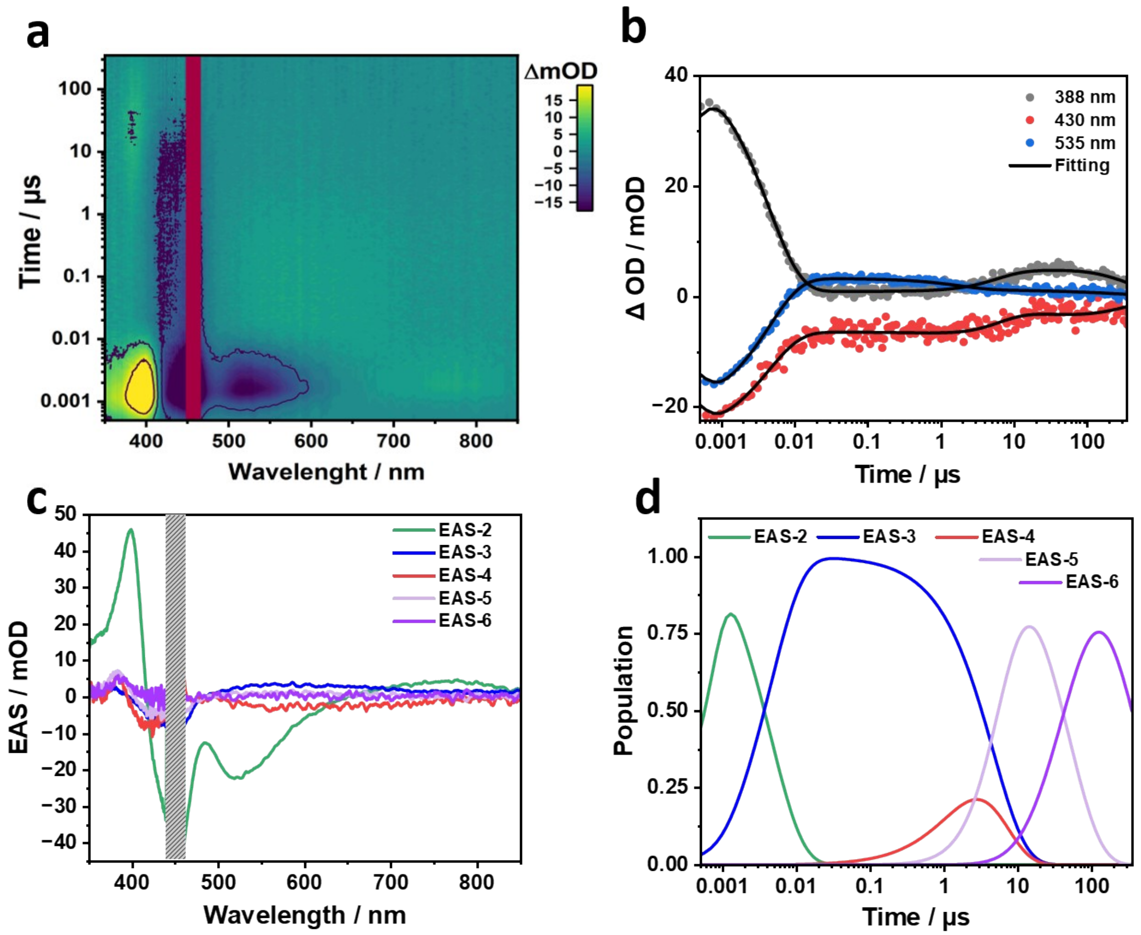

Figure S31 a, ns-TA heat map of 410 μM **(Pf-H)**$^+$ in aqueous solutions with 4.28 vol% TEOA and 0.55 M KH_2PO_4. The photoexcitation wavelength was set to 450 nm. **b**, Representative time traces at 388, 430, and 535 nm depicting the recorded evolution of the transients. **c**, Evolution associated spectra (EAS) from the sequential five-exponential global analyses of the ns-TA spectra. EAS-2 and EAS-3 correspond to (S_1^{rel})**(Pf-H)**$^+$ with a lifetime of 4.5 ns and (T_1)**(Pf-H)**$^+$ with a lifetime of 4.6 μs. EAS-4 and EAS-5 possess features of both **(Pf-H)**$^\bullet$ and **(Pf-H$_2$)**$^{\bullet+}$ with lifetimes of 6.5 and 48.8 μs, respectively. EAS-6 corresponds to **(Pf-H$_2$)**$^{\bullet+}$/**(Pf-H$_2$)**$^{\bullet+}$. **d**, Time profiles depicting the time-resolved population of the corresponding EASs from global analyses of the ns-TA spectra.

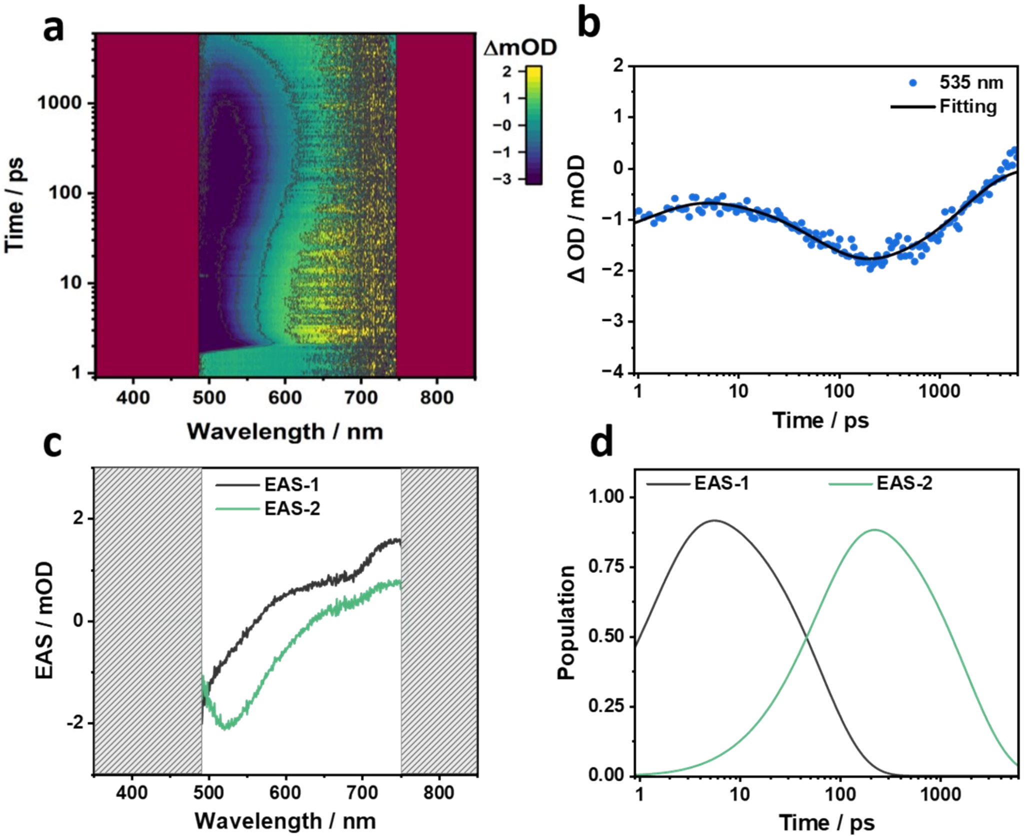

Figure S32. a, fs-TA heat map of 1000 μM **(Pf-H)**$^+$ in aqueous solutions with 4.28 vol% TEOA, 0.55 M KH_2PO_4, and purged with CO_2. The photoexcitation wavelength was set to 450 nm. **b**, Representative time trace at 535 nm depicting the recorded evolution of the transients. **c**, Evolution associated spectra (EAS) from sequential two-exponential global analyses of the fs-TA spectra: EAS-1 and EAS-2 correspond to (S_1^{hot})**(Pf-H)**$^+$ with a lifetime of 63 ps and (S_1^{rel})**(Pf-H)**$^+$ with a lifetime of 3.6 ns, respectively. **d**, Time profiles depicting the time-resolved population of the corresponding EASs from global analyses of the fs-TA spectra.

[1] J. J. Snellenburg, S. Laptenok, R. Seger, K. M. Mullen, I. H. M. van Stokkum, *J. Stat. Softw.* **2012**, *49*, 1 - 22.
[2] T.B. Coplen, *Rapid Commun. Mass Spectrom*. **2011**, *25*, 2538-2560.
[3] G. St-Jean, *Rapid Commun. Mass Spectrom.* **2003**, *17*, 419-428.
[4] R. van Geldern, M. P. Verma, M. C. Carvalho, F. Grassa, A. Delgado-Huertas, G. Monvoisin, J. A. C. Barth, *Rapid Commun. Mass Spectrom*. **2013**, *27*, 2099-107.
